# Stable Cortical Body Maps Before and After Arm Amputation

**DOI:** 10.1101/2023.12.13.571314

**Published:** 2023-12-14

**Authors:** Hunter R. Schone, Roni O. Maimon Mor, Mathew Kollamkulam, Craig Gerrand, Alexander Woollard, Norbert V. Kang, Chris I. Baker, Tamar R. Makin

**Affiliations:** 1 Institute of Cognitive Neuroscience, University College London, London, UK; 2 Laboratory of Brain & Cognition, National Institutes of Mental Health, National Institutes of Health, Bethesda, Maryland, USA; 3 Rehab Neural Engineering Labs, University of Pittsburgh, Pittsburgh, PA, USA; 4 Department of Physical Medicine and Rehabilitation, University of Pittsburgh, Pittsburgh, PA, USA; 5 Department of Experimental Psychology, University College London, London, UK; 6 UCL Institute of Ophthalmology, University College London, London, UK; 7 Department of Experimental Psychology, University of Oxford, Oxford, UK; 8 Department of Orthopaedic Oncology, Royal National Orthopaedic Hospital NHS Trust, Stanmore, Middlesex, UK; 9 Plastic Surgery Department, Royal Free Hospital NHS Trust, London, UK; 10 Wellcome Centre for Human Neuroimaging, UCL Institute of Neurology, London, UK; 11 MRC Cognition and Brain Sciences Unit, University of Cambridge, Cambridge, UK

## Abstract

Neuroscientists have long debated the adult brain’s capacity to reorganize itself in response to injury. A driving model for studying plasticity has been limb amputation. For decades, it was believed that amputation triggers large-scale reorganization of cortical body resources. However, these studies have relied on cross-sectional observations post-amputation, without directly tracking neural changes. Here, we longitudinally followed adult patients with planned arm amputations and measured hand and face representations, before and after amputation. By interrogating the representational structure elicited from movements of the hand (pre-amputation) and phantom hand (post-amputation), we demonstrate that hand representation is unaltered. Further, we observed no evidence for lower face (lip) reorganization into the deprived hand region. Collectively, our findings provide direct and decisive evidence that amputation does not trigger large-scale cortical reorganization.

## Introduction

Understanding the brain’s capacity for reorganization in response to trauma, such as when it loses a key source of input following arm amputation, remains a crucial frontier in basic and clinical neuroscience. Foundational research in non-human primates reported that following amputation or deafferentation of a body-part, the region of primary somatosensory cortex (S1) associated with the amputated limb, becomes responsive to inputs from neighboring body-parts (i.e., adjacent fingers or face; (1, 2). This has been used as evidence for both a collapse of the original representation of the amputated body-part and a reorganization of the adjacent cortical resources, hijacking the deprived territory (3). However, this longstanding view is increasingly being called into question by emerging evidence suggesting the preservation of neural representations of amputated limbs, as indicated by recent fMRI studies involving phantom limb movement. For example, by asking human amputees to voluntarily move their phantom fingers, researchers found that the neural representation for the missing hand can still be engaged and contains shared features with S1 hand representation of able-bodied controls (4–9). These findings suggest that the neural structure for the amputated limb is preserved, at least to some degree, despite amputation.

The dichotomy of these findings has led to starkly different perspectives. On one side, some researchers have called for a revision of the textbooks – plasticity is incremental and, as such, extensive brain reorganization is outdated (10, 11). On the other, some maintain that the two views are not conceptually exclusive – preservation and reorganization can co-exist (12–14). Indeed, most scientists still consider reorganization as a profound driver of rehabilitation (15–17). This debate has been hampered by an over-reliance on cross-sectional research methodologies, in both animal and human studies. While informative, cross-sectional designs are not ideally suited to directly address the research question – to what extent are the representations of the hand and its cortical neighbor, the face, impacted by amputation?

**Figure 1.**
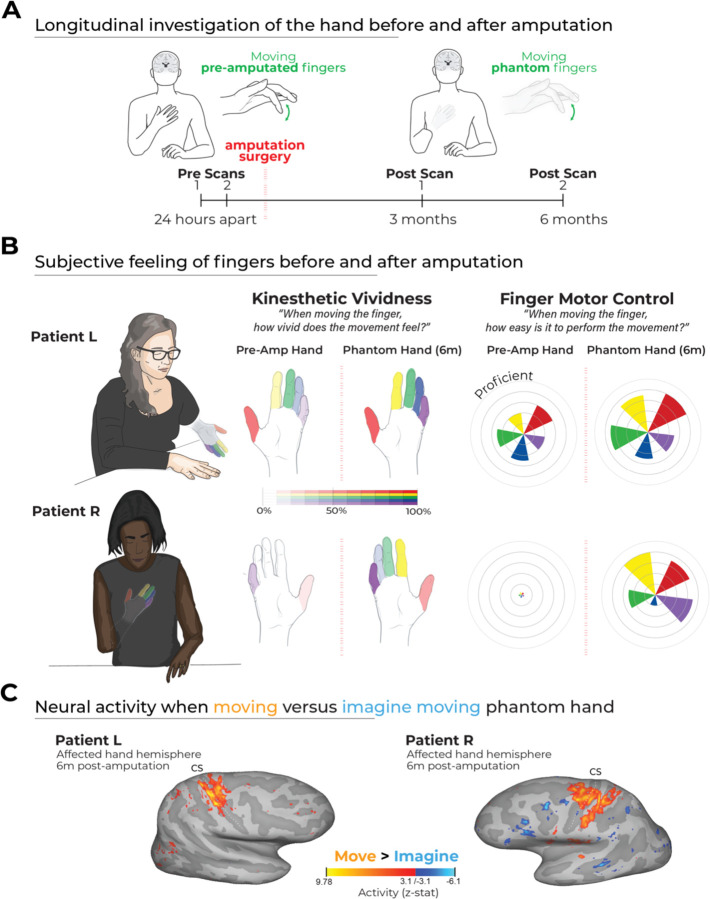
Longitudinal investigation of patients with planned hand amputations. **(A)** Experimental timeline. Pre- and post-amputation scans were conducted at four time points: twice (24 hours apart) before, and at 3 and 6 months after amputation. **(B)** Sensory experiences. Both patients reported increased vividness and motor control for the phantom fingers, relative to the pre-amputated fingers. Left – An illustration of the phantom limb position as reported by each patient (see Table 1). Middle - Kinesthetic vividness rated on a scale from 0 (no sensation) to 100 (as vivid as the unaffected hand) with color intensity indicating level. Right - Movement difficulty rated from 100 (as easy as the unimpaired hand) to 0 (extremely difficult). Finger colors: red=D1, yellow=D2, green=D3, blue=D4, purple=D5 (palm excluded). **(C)** Phantom movements are not imaginary. Univariate activity (z-scored) contrast map displaying patient attempts to move the phantom hand vs. imagining movement, 6 months post-amputation.

To bridge this gap, our research implemented a critical, longitudinal approach to follow the cortical representations of the hand and lower face (lips) in two adult patients before and up to 6 months following arm amputation (Video 1), as compared with a cohort of control participants over a similar timeframe (Figure 1A). By investigating changes longitudinally, within the same subject, this experimental approach can provide unique insights into any changes of somatosensory representation, without the confounding effects of cross-sectional comparisons (18). Our extensive investigation revealed no evidence for any large-scale plasticity in the representation of the body, either for the removed body part (fingers) or the remaining body parts (lips). Collectively, our findings provide direct and decisive evidence that amputation does not trigger large-scale cortical reorganization.

## Results

To investigate any changes in body representations, we measured hand and lip representations in two adult patients scheduled for an arm amputation, due to a vascular malformation (Patient L) and cancer (Patient R; demographics summarized in Table 1), as well as 16 able-bodied controls over 4 timepoints across 6 months (Figure 1A). We first examined the subjective feeling of moving the missing (phantom) fingers versus the fingers, pre-amputation. We then compared attempted phantom hand movements versus imaginary hand movements. We next investigated hand and finger topography along S1 using univariate activity. Then, we compared intra- and inter-finger representational content and structure in the S1 hand area using several multivariate approaches. Finally, to test whether adjacent body representations reorganize into the hand region, we quantified lip activity in the hand region, as well as the spatial topography of the lips relative to the hand.

Before undergoing amputation, both patients were able to move all fingers: Patient L exhibited proficient finger individuation, while Patient R showed a limited range of movement with notable enslavement between fingers (see Supp. Video 1 of patient’s pre-amputation motor control). Post-amputation, both patients reported highly vivid phantom limb sensations, including the ability to move individual phantom fingers (summarized in Figure 1B). Interestingly, both patients reported to feel their phantom finger sensations more vividly and were easier to move than their fingers pre-amputation. Importantly, while following instructions to move the phantom fingers, both patients produced muscle contractions in the residual limb confirming that they were attempting to move rather than just imagining the movement (Supp. Video 1). This was further demonstrated by the selective activation of sensorimotor brain regions by both patients when comparing attempted versus imagined phantom movements (Figure 1C). Combined, it appears both patients can attempt to move individual phantom fingers and the fMRI activity of these movements seem to be reflective of hand motor control (i.e., not imaginary), though it’s not yet clear whether this activity reflects the pre-existing hand.

### Stable hand and finger topography before and after amputation

**Figure 2.**
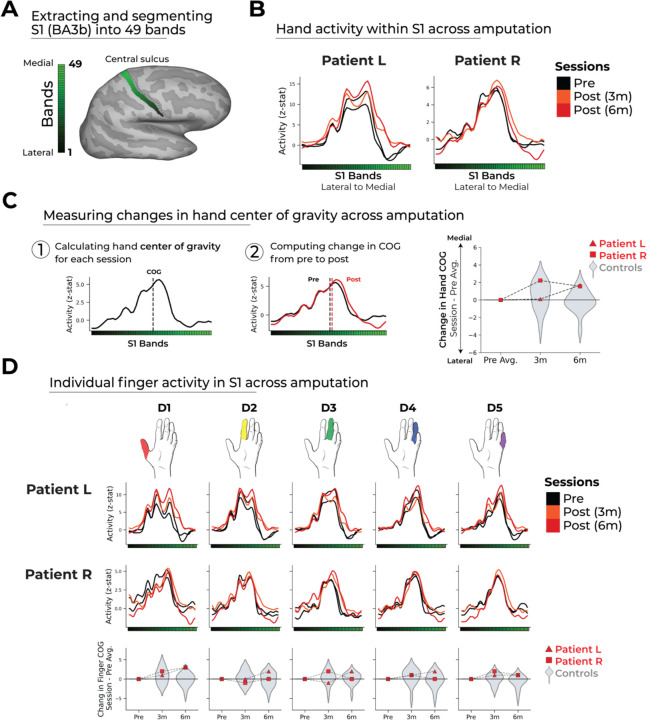
Stable hand and finger topography in the affected hemisphere despite amputation. (A) S1 (BA3b) region of interest (ROI). Estimated BA3b segmentation into 49 bands of similar height. (B) Longitudinal hand activity (versus rest) projected across the ROI. Affected hand's activity over four sessions (indicated in the legend). Black lines reflect pre-amputation activity, orange/red lines post-amputation. (C) Centre of Gravity (CoG) analysis. (1–2) calculated spatial shift of hand activity in BA3b pre- and post-amputation. Right – Shifts in post-amputation hand activity CoG in patients (red) does not deviate from inter-session variations found controls (gray). (D) Finger-Specific Activity. Top/middle rows show projected patients longitudinal activity profiles across BA3b ROI for each finger; bottom row shows finger CoG shifts before and after amputation. Positive values indicate medial shifts (toward feet), negative values lateral (toward lips) in BA3b. Patients CoG shifts for individual fingers fell within the distribution of controls. Values indicate group means ± standard error. Control data shown as gray violin plots. Patient L data shown as a red triangle and Patient L data shown as a red square. For simplicity, the control values are all for the left (non-dominant) hand.

During scanning, participants were visually cued to perform movements of individual fingers, lips, and feet. Our primary analyses focus on S1, specifically Broadman Area 3b (BA3b), known for its detailed topography and implication with post-amputation cortical reorganization (1, 2). Using an atlas-defined BA3b ROI (19), we segmented the entire strip of BA3b into 49 bands, which were registered to the brains of individual subjects (Figure 2A; see Methods). We then projected the univariate fMRI activity for the hand (averaged over all fingers versus rest) along these S1 bands for each hemisphere, session, and participant.

Both patients exhibited strikingly consistent hand activity before and after amputation, such that spatial activation for the phantom hand did not qualitatively differ from the pre-amputated hand activity in terms of either amplitude or spread across S1 (Figure 2B). To quantify changes in this topographic activity spread, we calculated the center of gravity (CoG) for the hand’s activity projection (Figure 2C). Both patients showed a stable hand CoG following amputation, such that any pre to post session differences in patient’s hand CoG were not significantly different from able-bodied controls (Figure 2C; two-tailed Crawford and Howell t-tests: Patient L: 3m: t(15)=−0.33, p=0.74; 6m: t(15)=1.44, p=0.17; Patient R: 3m: t(15)=0.22, p=0.82; 6m: t(15)=1.33, p=0.20; see Supp. Figure 1 for stable hand activity magnitude). Notably, this stability cannot be attributed to a pre-existing baseline (pre-amputation) difference existing between either patient and the controls (hand CoG; two-tailed Crawford and Howell t-test: Patient L: t(15)=−0.90, p=0.38; Patient R: t(15)=−1.03, p=0.31), indicating a normal hand activity profile both before and after amputation. Therefore, amputation does not trigger a large-scale disruption of hand activity at this relatively broad scale.

Next, we investigated the hand map in greater detail, examining activity for individual fingers, using the same topographic approach (Figure 2D). In both patients, despite not having a physical hand, the idiosyncratic activity pattern for each finger across our S1 region of interest was qualitatively stable before and after amputation in magnitude and spread. Both patients showed a stable CoG for each finger following amputation, such that any pre to post session differences in patient’s finger CoG were not significantly different from controls (two-tailed Crawford and Howell t-tests: Patient L (6m): 0.64 ≤ *p* ≤ 1.0; Patient R (6m): 0.64 ≤ *p* ≤ 1.0; FDR correction for multiple comparisons). Similar stability was also observed for the patient’s intact (unaffected) hand (Supp. Figure 2A–B) and individual fingers (Supp. Figure 2C). Collectively, our findings provide clear evidence for the existence of stable topographic hand and finger representations despite amputation.

### Stable intra-finger representational structure before and after amputation

**Figure 3.**
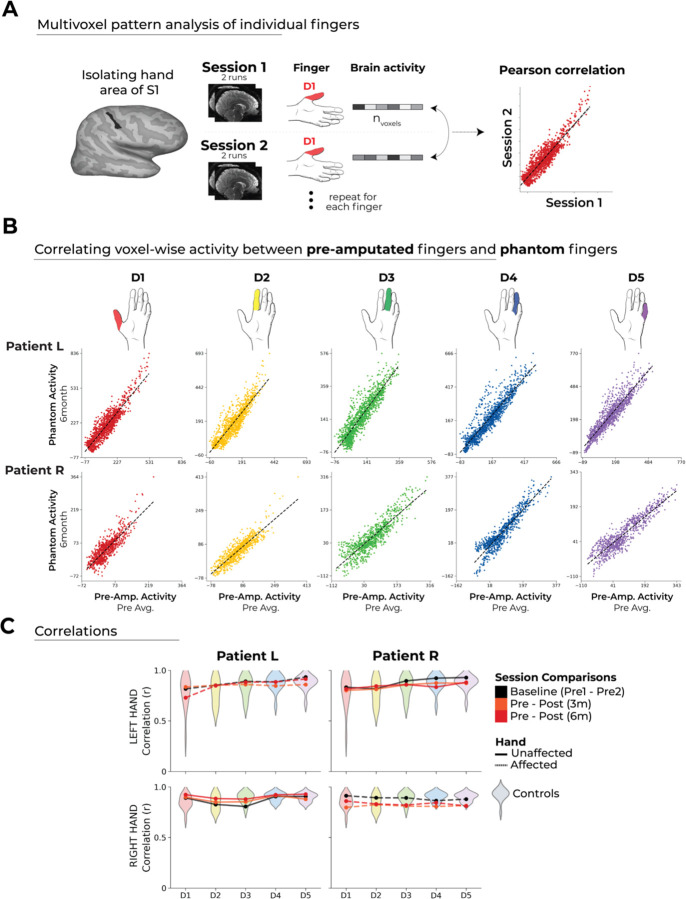
Correlating pre- to post-amputation multivoxel finger activity patterns. (A) Inter-session Pearson correlations of individual fingers within the BA3b hand region. (B) Example Correlation Visualization: For each finger of the patients, voxel-wise activity correlations before and after amputation are shown. Panel B represents the first permutation [Pre Avg. Runs 1 and 2 versus Post (6m) Runs 1 and 2] of the correlations that are comprehensively performed in panel C. (C) Inter session correlations for the left (top row) and right hands (bottom) in the contralateral hand ROI. Line colours indicate session pairings (indicated in the legend). For patients, dashed line denotes the affected hand; solid line unaffected hand. Violin plots reflect able-bodied control’s Pre – Post (6m) values. All between and within-session correlations are depicted in Supp. Figure 3. All other annotations are as in Figure 2.

One possibility is that our initial approach may not be sensitive enough to capture changes at a more fine-grained scale because it focused on broader topographic changes. Therefore, we implemented a multi-voxel pattern approach within a more narrowly defined region of interest (ROI), specifically the hand area of S1 (Figure 3A; see Methods). By performing cross-session Pearson correlations of activity patterns when moving each finger, we aimed to directly compare the stability of these representations before and after amputation. In both patients, we observed very high correlations when comparing activity patterns of pre-amputated fingers (Pre) to phantom fingers (6m) [Figure 3B; Patient L (Pre to 6m): range of finger correlations: 0.72 ≤ *r* ≤ .91, p < 0.0001; Patient R (Pre to 6m): 0.82 ≤ *r* ≤ .86, p < .0001]. This direct pre-post comparison effectively demonstrates that pre-amputation finger activity is strongly significantly associated with phantom finger activity post-amputation.

While we observed significant pre to post correlations for each finger in the patients, we next tested whether these pre to post correlation coefficients are worse relative to able-bodied controls. We found that the patient’s pre to post correlation coefficients fell within the typical variability range seen in the controls (Figure 3C; two-tailed Crawford and Howell t-test: 0.518 ≤ *p* ≤ 0.969; FDR correction for multiple comparisons; for able-bodied controls’ correlation values over time see Supp. Table 1). In other words, amputation does not trigger unique changes in the multivoxel activity patterns for individual fingers, relative to the typical stability of these patterns in able-bodied controls.

### Stable inter-finger structure before and after amputation

**Figure 4.**
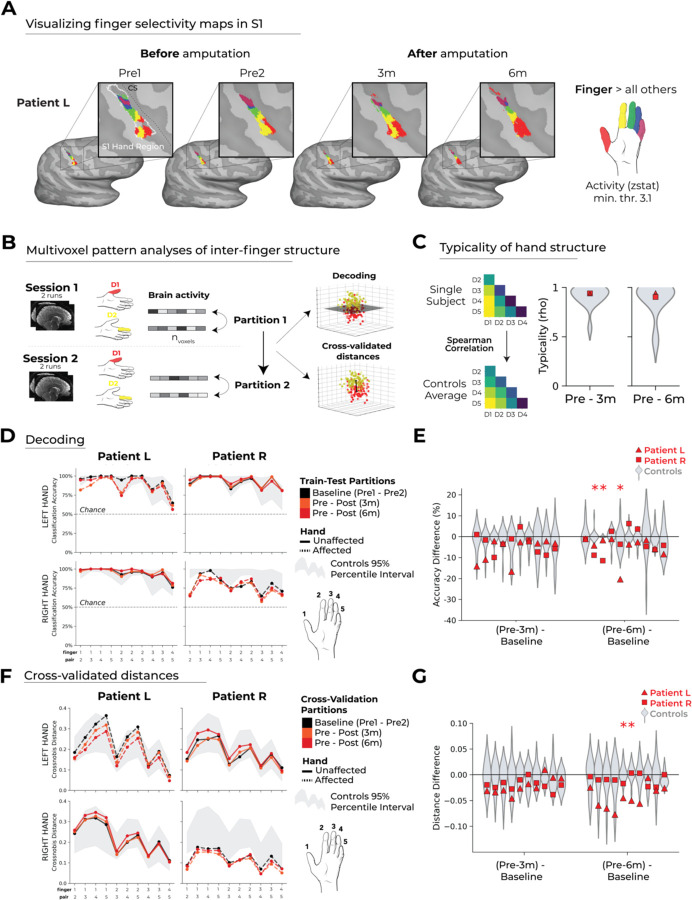
Inter-Finger Representational Consistency Pre- and Post-Amputation. (A) Finger selectivity maps for Patient L. Thresholded (Z>3.1) contrast maps for each finger (versus all others), masked to the hand ROI. Color codes indicated on the right. Patient R’s data did not reach the necessary threshold for this visualization pre-amputation (Z > 3.1), possibly due to less independent finger movement control (Supp. Video 1). (B) Graphic illustration of multivoxel pattern analyses. (C) The representational typicality of the hand structure was estimated by correlating each session’s cross-validated mahalanobis distances for each individual to a canonical inter-finger structure (controls average). Inter-finger multivariate analysis using (D-E) linear SVM decoder and (F-G) cross-validated mahalanobis (cross-nobis) distances. Line colours denote train-test/cross validation session pairs, respectively as indicated in the legend. The gray shaded area reflects able-bodied control’s Pre – Post (6m) data (95% percentile interval). (D, F) Classification/distance differences before and after amputation are visualized for each finger pair [Pre Avg. – Post1 (3m)] minus [Pre1-Pre2] and [Pre Avg. – Post2 (6m)] minus [Pre1-Pre2]. Each violin plot reflects an individual finger pair (same order of finger-pairs as detailed in C-E). * denotes significance of Crawford test for at least 1 patient (p < .05, FDR corrected for multiple comparisons). All other annotations are the same as described in Figure 3.

In the previous section, we observed that the multivoxel activity patterns for individual fingers were stable before and after amputation. Though, it is possible that, while information about each finger is relatively stable after amputation, the selectivity of individual fingers (relative to each other) could be degrading (9). Mapping finger selectivity (each finger versus all others) as demonstrated for Patient L in Figure 4A may be too crude of a measure for this purpose (as demonstrated in (20). To quantify more fine-grained changes in this inter-finger representational structure, we next applied multivoxel pattern analyses using a linear support vector machine classifier (Figure 4B). By training the classifier on data from one session (e.g., pre-amputation) and testing on data from another session (e.g., post-amputation), this approach allowed us to determine the extent to which finger selective information remains consistent before and after amputation.

While both patients exhibited significant pre to post decoding, we did observe, at baseline (pre-amputation), significantly lower classification for the pre-amputated hand of Patient R (two-tailed Crawford and Howell t-test: t(15)=−5.66, *p* < 0.001) and not Patient L (two-tailed Crawford and Howell t-test: t(15)=−0.31, p = 0.75; Supp. Figure 4). This discrepancy may be due to Patient R’s impaired ability to perform the task with their affected hand pre-amputation (Supp. Video 1). Regardless of the baseline difference, the primary question here is whether a pre-amputation trained classifier could categorize phantom finger movements and vice versus.

When training the classifier on the pre-amputation data and testing it on the post-amputation data (and vice versus), both patients exhibited significantly above chance pre-to-post classification accuracies [Figure 4D; one-sample t-test: Patient L: Pre/6m: 87%; p < 0.001; Patient R: Pre/6m: 76%; p < 0.001]. This provides direct evidence that the representational finger information prior to amputation remains accessible post-amputation.

We next tested whether the finger selective information uniquely decreases in the patients pre to post session comparisons, relative to the baseline pre-amputation comparisons. While most finger pair-wise comparisons did not significantly differ compared to controls, we indeed observed significant decreases in accuracy for 2 specific finger pairs (out of 20 comparisons per patient), indicating a slight reduction in finger-specific information post-amputation [Figure 4E; two-tailed Crawford and Howell t-test: Patient L (6m): D1-D4, t(15) =−4.08, p = 0.02; D2-D3: t(15)=−3.56, p = 0.03; Patient R (6m): D1-D3: t(15) = −3.65, p = 0.02; D1-D4: t(15)=−27.8, p < 0.001; FDR correction for multiple comparisons], which were not observed for the intact hand (Supp. Figure 6A). In other words, there appears to be a slight decrease in finger selective information following amputation.

Considering the decoding analysis neared ceiling performance (close to 100%), we also performed a representational similarity analysis using cross-validated mahalanobis distances (a non-binary, continuous measure) across sessions. All between and within-session mahalanobis distance values are depicted in Supp. Figure 5. This analysis too confirmed significant finger selective information existing for both patients following amputation, as demonstrated using a one-sample comparison of the finger pairwise distances against zero (Figure 4F; Patient L (Pre to 6m) affected hand: t(9)=7.60, p < 0.0001; intact hand: t(9)=8.70, p < 0.0001; Patient R (Pre to 6m) affected hand: t(9)=8.57, p < .0001; intact hand: t(9)=9.51, p < .0001). While no significant pre-post differences were observed for Patient R, Patient L showed a reduction in 2/20 finger pair distances (both at the 6 month session), as compared to the able-bodied controls, indicating some reduction in D2 selectivity post-amputation (Figure 4G; two-tailed Crawford and Howell t-test: D2-D3: t(15)=−3.37, p = 0.004; D2-D4: t(15) = −3.74, p = 0.002; FDR correction for multiple comparisons). For context, when examining these differences for the intact hand, we qualitatively observed an opposite trend with distances increasing for both patients, though no finger pair reached significance relative to controls (Supp. Figure 6B). Thus, across two multivariate analyses, we observed largely stable finger selective information post-amputation, with a slight, though inconsistent, decrease in finger selective information for a few finger pairs.

More than just the magnitude of finger selective information for single finger pairs, the neural signature of a hand representation is indexed as the overall pattern across inter-finger distances (i.e., the hand representational structure). We therefore tested whether the inter-finger distance pattern was typical (or normal) in the patients by correlating each participant’s inter-finger distance pattern with a canonical inter-finger distance pattern. From this analysis, we observed that both patient’s typicality scores showed no deterioration following amputation, as compared to the able-bodied controls (two-tailed Crawford and Howell t-test: Patient L: 0.66 ≤ *p* ≤ 0.80; Patient R: 0.80 ≤ *p* ≤ 0.94; two-tailed; Figure 4C), thus demonstrating that any slight reductions in selectivity observed in the pairwise comparison above, were not sufficient to impact the preservation of the representational structure post-amputation. Collectively, these analyses confirm that despite a small decrease in the magnitude of finger selectivity, the overall information content and typicality of the representational structure, following amputation, is largely preserved.

### No evidence for large-scale reorganization of the lips into the deprived hand territory

From the previous analyses, it is clear the hand representation is stable before and after amputation, but what about reorganization of the lip representation, previously implicated with reorganization following arm amputation? As a final analysis, we investigated whether there is any evidence to support a large-scale shift of the lip representation into the deprived hand territory (2, 3). When projecting the fMRI activity for the hand and lips onto the S1 bands, there was no evidence in either patient for a shift of the lip representation towards the hand region post-amputation (Figure 4). Indeed, both patients showed typical longitudinal changes within the range observed in control participants in both the CoG for the lip representation (Figure 4E; two-tailed Crawford and Howell t-test: Patient L: 3month: t(15)=−0.40, p=0.69; 6month: t(15) = 0.02, p=0.97; Patient R: 3m: t(15)=−0.89, p=0.38; 6month: t(15) = −0.98, p=0.33) and the average lip activity within the hand region of S1 (Figure 4G; two-tailed Crawford and Howell t-test: Patient L: 3month: t(15) = −0.05, p=0.95; 6month: t(15) = 1.04, p=0.31; Patient R: 3month: t(15) = 0.68, p = 0.50; 6month: t(15) = 0.70, p=0.49). This lack of lip reorganization was further consolidated when considering similar cross-session variations in the unaffected hemisphere (Supp. Figure 7). That is to say, amputation does not impact the lips topographic spread and overall magnitude across S1 and specifically within the hand region. Collectively, these results show that lip topography in S1 is highly stable across time and amputation, with no evidence to suggest amputation triggers large-scale reorganization of the lips into the hand region.

## Discussion

While previous studies have observed that cortical hand representations in amputees mirror those of able-bodied individuals (4, 5, 9, 21), our longitudinal study unveils a deeper truth: the neural finger-print of the hand pre-amputation is undeniably preserved in the brain's functional architecture. We observed strikingly consistent activity, both in spread and magnitude, for the hand and individual fingers. The pre-amputation multivoxel activity patterns of fingers significantly correlated with the phantom fingers. Perhaps most strikingly, we can decode the representation of the missing fingers from the pre-amputated hand representation. While we observed a partial decrease in the magnitude of finger selectivity, the overall information content and typicality of the representational structure, following amputation, remained intact. Finally, we observed no evidence for changes in lip activity – both in terms of activity spread along S1 and magnitude within the S1 hand area. Collectively, our findings reveal a remarkable stability in the brain's representation of the hand, individual fingers, and the relationships between them, despite the loss of the limb.

Synthesizing the breadth of neuroscience research on this topic reveals that our findings are not isolated, but rather align with a much broader spectrum of evidence indicating the absence of cortical reorganization. For example, longitudinal mouse studies demonstrate that shifts in single-cell input preferences, due to deprivation or lesions, reflect the strengthening of existing neural connections, rather than structural reorganization (22) and maintained overall network connectivity (23). Viewed in this light, a multitude of evidence across various modalities is mounting, from the development of right-lateralized language abilities in children with perinatal stroke, to visual cortex remapping due to retinal lesions (24). The revised view to these and other canonical examples proposes that the supposedly dramatic change in receptive fields (25–27) actually underscores relatively minor, and potentially top down modulation of existing neural networks (28, 29) rather than a categorical remapping of cortical functions (11). Cochlear implants for congenital deafness (30), sight restoration for early blindness [see (31)], and hand transplantation in amputees (32), all suggest the preservation of native processing in cortical areas deprived of their natural inputs. Therefore, the deprived cortex has not been ‘taken over’ by an alternative function [see (20) for a critical re-evaluation of the classical reorganization findings following amputation]. Our longitudinal research further revises this perspective, and other related cross-sectional studies (5, 9, 21, 33), by showing that, even at the meso and macro scale, there's no significant upregulation or alteration to local processing following primary input loss— whether it pertains to the hand, individual fingers, or lip activity. Contrary to recent views (34), we don’t find clear evidence for plasticity, neither Hebbian or homeostatic. The stability of our activity profiles, from univariate magnitude and spread, to multivariate correlations and decoding, argues decisively against the theory of widespread cortical reorganization following amputation. Instead, our findings suggest that the brain maintains its pre-existing functional integrity, to the extent that it does not alter the unique representational fingerprint of the pre-amputated hand.

While our results may challenge established neuroscience perspectives, they strongly resonate with patients' experiences – who continue to experience their hands as phantoms following amputation (35). When further considering the clinical circumstances of each patient, there are unique factors that help to contextualize their results. For instance, while reporting a substantially improved sense of movement in her phantom fingers, we observed an unchanged neural hand representation for Patient R. In contrast, despite a drastic change in the nature of the nerve’s input to S1, including targeted muscle reinnervation (36) and regenerative peripheral nerve interfaces [(37); Supp. Figure 8], patient L’s hand representation also remained stable. Despite having very different chronic phantom limb pain experiences, both participants showed relatively similar hand stability. Combined, these cases indicate that a limb's pre-amputation state, rather than post-amputation neural changes, is crucial for understanding the post-amputation brain representation.

We must consider potential limitations in our study. First, had we extended our study beyond 6 months post-amputation, would we have observed more changes? Some of the original non-human primate studies on S1 reorganization based their findings at comparable (and indeed shorter) timescales (1, 38, 39). Furthermore our prior cross-sectional research demonstrated that even 30 years post-amputation, a representation of the missing hand can be found (5). Second, we only tested 2 patients. This was due to the immense difficulty of recruiting participants to the study, considering the very unique inclusion and exclusion criteria (e.g., preparing for an upper-limb amputation, ability to move individual fingers and MRI safe), and the relative high-density of testing. With most upper-limb amputations being unexpected (40) and the narrow pre-amputation window posing recruitment challenges, understandably, previous longitudinal designs in humans aimed at studying reorganization of the stump and/or face representations following amputation have been scarce and those that exist did not measure persistent (phantom) representations (41–43). This is why, out of eighteen potential participants over a seven-year recruitment window, we managed to study two patients, providing a case-to-case replication. The third limitation is our reliance on null results to support some of our conclusions, given the lack of robust statistical approaches for interpreting nonsignificant results (no consensus on a Crawford Bayesian test). While we agree that null results should be interpreted with caution, our results go beyond simply noting no differences relative to controls; we demonstrate that pre-amputation finger activity is highly associated with phantom finger activity. Further, we show that a classifier trained on data pre-amputation can significantly classify phantom fingers. As such, our key findings rest on positive (significant) findings.

Our study’s longitudinal lens carries significant implications for the field of brain-computer interface (BCI) development and phantom limb pain (PLP) treatments. For BCI research, our findings demonstrate that the brain’s highly detailed representations of amputated limbs can be harnessed immediately post-amputation and remain stable over time for long-term BCI applications. This establishes a foundation for crafting biomimetic-inspired (44) and robust neuroprosthetics. Our study further emphatically counters critiques that intracortical data from spinal cord injury (SCI) patients may not reflect the functioning of a healthy adult brain, due to proposed preexisting injury-induced remapping and reorganization (see Discussion sections of 46–49). Regarding PLP, our study offers a critical perspective on the neural mechanisms by which targeted muscle reinnervation and regenerative peripheral nerve interfaces operate. Contrary to current views (7, 49, 50), our findings indicate that these interventions do not appear to significantly alter the cortical representation of the hand. Therefore, the success of such interventions might not hinge on their ability to reshape cortical maps, as previously thought. Finally, and perhaps most importantly, our findings affirm the unaltered nature of sensory body maps following amputation, indicating any roles of both Hebbian and homeostatic plasticity in maintaining functional somatotopic organization are even more marginal than considered by even the field’s strongest opponents of large-scale reorganization (11, 24).

## Methods

### Participants

#### Patients

Over a 7-year period and across multiple NHS sites in the UK, we recruited 18 potential patients preparing to undergo hand amputations. Due to a multitude of factors (e.g., MRI safety contraindications, no hand motor control, age outside ethics, high level of disability), we could only perform pre-amputation testing on 6 patients. Due to additional factors (complications during surgery, general health, retractions) we successfully completed our full testing procedure on 2 patients (for patient demographics see Table 1).

#### Patient Amputation Surgeries

For the two patients reported here, there are noteworthy differences in their amputation surgeries. Patient L underwent an amputation to combat a rapidly developing arteriovenous malformation (AVM) in the upper arm. Before amputation, she had a relatively high level of motor control in the pre-amputated hand. Additionally, Patient L’s amputation included more advanced surgical techniques, involving a combination of targeted muscle reinnervation [TMR (36)] and regenerative peripheral nerve interfaces [RPNI; (37)]. In these approaches, rather than simply cutting the residual nerve, the remaining nerves were sutured to a new muscle (TMR) or implanted with a nerve graft near a new muscle target (RPNI; in Patient L’s case, the technique varied depending on the muscle, see Supp. Figure 8). Patient R underwent a traditional amputation procedure to remove a sarcoma tumor that had been slowly progressing since 1995. The multiple operations of the arm, prior to the amputation, left her with restricted motor control of the fingers, though still able to move them (see Supp. Video 1).

#### Able-bodied control group

In addition to the patients, we tested a control group which included 16 older able-bodied participants [9 females; mean age ± std = 53.1 ± 6.37; all right-handed]. The control group also completed four fMRI sessions at the same timescale as both patients and were age-matched to Patient R. 4 additional controls were also recruited for this group, however, we did not complete their testing, due to retractions and incidental findings captured in the MRI sessions.

Ethical approval was granted by the NHS National Research Ethics Committee (18/LO/0474), and in accordance with the Declaration of Helsinki. Written informed consent was obtained from all participants prior to the study for their participation, data storage and dissemination.

One additional consideration is that Patient L is much younger than the control group. We pooled a dataset from a previous study of 32 able-bodied controls of a similar age to Patient L (mean ± STD: 23.1 ± 3.89), each were scanned twice, one-week-apart on the same fMRI task and scanner (51). However, because Patient L met our criteria for similar hand representation pre-amputation (Supp. Figure 4), this data was pooled, but not analyzed.

### Questionnaires

Due to a restricted time window for performing the tests before amputation, as well as the patients’ high level of physical discomfort and emotional distress, we were highly limited in the number of assessments we could perform. As such we focused the physically-involved testing on the functional neuroimaging tasks. However, in addition, we collected data on multiple questionnaires and had participants perform a functional ecological task.

#### Kinesthetic vividness

Kinesthetic vividness was quantified for each finger before and after the amputation [“*When moving this finger, how vivid does the movement feel? Please rate between 0 (I feel no finger movement) to 100 (I feel the finger movement as vividly as I can feel my other hand finger moving).”]*

#### Finger motor control

Perceived finger movement difficulty was quantified for each finger before and after amputation [“*When moving this finger, how difficult is it to perform the movement? Please rate between 100 (I found it as easy as moving the homologous finger in the unimpaired hand) to 0 (the most difficult thing imaginable).”].*

#### Pain ratings

Before and after amputation, both patients were asked to rate the frequency of their pre-amputation limb pain or post-amputation phantom limb pain, respectively, as experienced within the last year, as well as the intensity of worst pain experienced during the last week (or in a typical week involving pain; see Table 1). Chronic pain was calculated by dividing worst pain intensity (scale 0–100: ranging from no pain to worst pain imaginable) by pain frequency (1 – all the time, 2 – daily, 3 – weekly, 4 – several times per month, and 5 – once or less per month). This approach reflects the chronic aspect of pain as it combines both frequency and intensity (6, 52). A similar measure was obtained for non-painful phantom sensation vividness and stump pain. Both patients also filled out the Pain Detect questionnaire (53). Additionally, before and after amputation, both patients reported intensity values for different words describing different aspects of pain, quantified using an adapted version of the McGill Pain Questionnaire, (54). For each word, participants were asked to describe the intensity between 0 (non-existing) to 100 (excruciating pain) as it relates to each word. Please note that we used a larger response scale than standard to allow the patients to articulate even small differences in their pain experience (see Supp. Figure 9).

#### Functional Index

Before and after amputation, both patients were asked to rate their difficulty at performing a diversity of functional activities because of their upper limb problem, quantified using the Upper Extremity Functional Index (55).

### Ecological Task

To characterize habitual compensatory behavior, participants completed a task involving wrapping a present [based on (56)]. Task performance was video recorded but will not be reported in this paper.

### Scanning Procedures

Each MRI session consisted of a structural scan, four fMRI finger-mapping scans and two body localizer scans, which we report here.

### fMRI Task Design

#### Finger-mapping scans

The fMRI design was the same as a previous study from our lab (51), though specific adaptations were made to account for the phantom experience of the patients (described below). Considering that S1 topography is similarly activated by both passive touch and active movement (57), participants were instructed to perform visually cued movements of individual fingers, bilateral toe curling, lips pursing or resting (13 conditions total). The different movement conditions and rest (fixation) cue were presented in 9-second blocks and each repeated 4 times in each scan. Additionally, each task started with 7 seconds of rest (fixation) and ended with 9 seconds of rest.

To simulate a phantom-like tactile experience for the patient’s pre-amputation, the affected hand was physically slightly elevated during scanning such that affected finger tapping-like movements were performed in the air. Alternatively, for the unaffected hand (before and after amputation), the individual finger movements were performed in the form of button presses on an MRI-compatible button box (four buttons per box) secured on the subject’s thigh. The movement of the thumb was performed by tapping it against the wall of the button box. For the control participants, half of the participants had the right hand elevated, performing the finger movements in the air, and the other half had the left hand elevated.

Instructions were delivered via a visual display projected into the scanner bore. Ten vertical bars, representing the fingers, flashed individually in green at a frequency of 1 Hz, instructing movements of a specific finger at that rate. Feet and lips movements were cued by flashing the words “Feet” or “Lips” at the same rate. Each condition was repeated four times within each run in a semi-counterbalanced order. Participants performed four scan runs of this task. One control participant was only able to complete 3 runs of the task for one of the sessions.

#### Imagery control scans

In each of the two body localizer scans, participants were visually cued to move each hand, imagine moving the affected (patients) or non-dominant hand (controls), in addition to actual lips, toes (on the affected side only) and arm (on the affected side only) movements. The different movement conditions and a rest (fixation) cue were presented in 10-second blocks and repeated 4 times in each scan.

### MRI Data Acquisition

MRI images were obtained using a 3-Tesla Quattro scanner (Siemens, Erlangen, Germany) with a 32-channel head coil. Anatomical data were acquired using a T1-weighted magnetization prepared rapid acquisition gradient echo sequence (MPRAGE) with the parameters: TR = 2.53 s, TE = 3.34 ms, FOV = 256 mm, flip angle = 7°, and voxel size = 1 mm isotropic resolution. Functional data based on the blood oxygenation level-dependent signal were acquired using a multiband gradient echo-planar T2*-weighted pulse sequence (58) with the parameters: TR = 1.5 s, TE = 35 ms, flip-angle = 70°, multi-band acceleration factor = 4, FOV = 212 mm, matrix size of 106 x 106, and voxel size = 2 mm isotropic resolution. Seventy-two slices, with a slice thickness of 2 mm and no slice gap, were oriented parallel to the anterior commissure – posterior commissure, covering the whole cortex, with partial coverage of the cerebellum. Each of the four functional runs comprising the main task consisted of 335 volumes (8 min 22 s). Additionally, there were 204 volumes for the two imagery control scans (5 min 10 s). For all functional scans, the first dummy volume of every run was saved and later used as a reference for co-registration.

### fMRI Analysis

Functional MRI data processing was carried out using FMRIB’s Expert Analysis Tool (FEAT; Version 6.0), part of FSL (FMRIB’s Software Library, www.fmrib.ox.ac.uk/fsl), in combination with custom bash, Python (version 3) and Matlab scripts [(R2019b, v9.7, The Mathworks Inc, Natick, MA; including an RSA toolbox (59, 60). Cortical surface reconstructions were produced using FreeSurfer [v. 7.1.1; (61, 62)] and Connectome Workbench (humanconnectome.org) software. Decoding analyses were carried out using scikit-learn (v.1.2.2).

### fMRI Preprocessing

The following pre-statistical processing was applied: motion correction using MCFLIRT (63), non-brain removal using BET (64), spatial smoothing using a Gaussian kernel of FWHM 3mm for the functional task data, grand-mean intensity normalization of the entire 4D dataset by a single multiplicative factor, and high-pass temporal filtering (Gaussian-weighted least-squares straight line fitting, with σ = 90 s). Time-series statistical analysis was carried out using FILM with local autocorrelation correction (65). The time series model included trial onsets convolved with a double γ HRF function; six motion parameters were added as confound regressors. Indicator functions were added to model out single volumes identified to have excessive motion (>.9 mm). A separate regressor was used for each high motion volume (deviating more than .9mm from the mean position). For the finger mapping scans, the average number of outlier volumes for an individual scan, across all subjects, was 1.5 volumes.

To ensure all 4 sessions (Pre1, Pre2, 3m, 6m) were well aligned, for each participant, we calculated a structural mid-space between the structural images from each session, i.e., the average space in which the images are minimally reorientated (66). The functional data for each individual scan run within a session were then registered to this structural mid-space using FLIRT (63, 67)

### Low Level Task-Based Analysis

We applied a general linear model (GLM) using FMRI Expert Analysis Tool (FEAT) to each functional run. For the primary task, the movement of each finger/body-part (10 fingers, lips and feet – total of 12 conditions) was modeled against rest (fixation). To capture finger selectivity, the activity for each finger was also modelled as a contrast against the sum of the activity of all other fingers of the same hand.

We performed the same GLM analysis on the 6 conditions of the imagery scans. To capture the selectivity for actual attempted phantom movements versus imagine phantom hand movements, the activity for attempted hand movement was also modelled as a contrast against imagined hand movement.

For each participant, parameter estimates of the each of the different conditions (versus rest) and GLM residuals of all voxels were extracted from each run's first-level analysis. All analyses were performed with the functional data aligned to the structural mid-space.

### Regions of Interest

#### S1: Broadmann Area 3b

We were specifically interested in testing changes in topography within (and around) BA3b. First, the structural mid-space T1 image were used to reconstruct the pial and white-gray matter surfaces using FreeSurfer’s recon-all. Surface co-registration across hemispheres and participants was conducted using spherical alignment. Individual surfaces were nonlinearly fitted to a template surface, first in terms of the sulcal depth map and then in terms of the local curvature, resulting in an overlap of the fundus of the central sulcus across participants (68).

#### S1 (BA3b) hand region of interest

The BA3b ROI was defined in the fsaverage template space using probabilistic cytotectonic maps (68) by selecting all surface nodes with at least 25% probability of being part of the grey matter of BA3b (69). Further, for the multivoxel pattern analyses, we restricted the BA3b ROI to just the area roughly representing the hand. This was done by isolating all surface nodes 2.5 cm proximal/distal of the anatomical hand knob (70). An important consideration is that this ROI may not precisely reflect BA3b for each subject and may contain relevant activity from neighboring S1 areas, due to the nature of our data (3T fMRI, smoothing FWHM 3mm) and the probabilistic nature of the atlas. As such, we consider this as a definitive localizer of S1 and an indicative localizer of BA3b. The surface ROIs were then mapped to the individual volumetric high-resolution anatomy.

#### 49 bands of BA3b

To segment BA3b into 49 bands, we loaded the fsaverage cortical surface with the boundaries of the BA3b ROI, as defined by the Glasser atlas (19). We rotated the map so that the central sulcus was perpendicular to the axis. We overlayed a box with 49 bands of equal height, on this ROI. By masking the box to the ROI, we constructed 49 bands of the BA3b ROI. Because this masking approach requires drawing boundary lines using the vertices on the cortical flat map, we could optimally only get 49 bands (maximum) without issues with the boundary drawing approach. These ROIs were then mapped onto the individual subject cortical surfaces and further to the individual volumetric high-resolution anatomy.

### Univariate Activity (in the order the analyses are shown)

#### Patient contrast maps for moving versus imagine moving the phantom

To visualize the contrast maps for attempted versus imagine phantom hand movements, estimates from the two finger-mapping scan runs for each session were averaged in a voxel wise manner using a fixed effects model with a cluster forming z-threshold of 3.1 and family-wise error corrected cluster significance threshold of *p* < 0.05. These contrast maps are visualized in Figure 1C with a minimum z-threshold in both directions of 3.1.

#### Hand topography across 49 bands of BA3b

Using the 49 bands of BA3b (described above), we projected the neural activity for the hand (versus rest) for each hemisphere (contralateral to the hand being moved), session and participant. The average activity across all voxels within each band was averaged to extract a single value per band.

#### Center of gravity

To quantify changes in the hand, finger or lip topography, we computed the center of gravity (CoG) of activity (for a single body-part) across the 49 BA3b bands. To do this, we first computed the weighted activity (β_w_) across the bands. To do this each band number was multiplied by the average activity in the band.


$$\beta_{w}=\left(1\;\;x\;\beta_{1}\right)+\left(2\;\;x\;\beta_{1}\right)\ldots$$

To compute the CoG, we then divided the sum of the weighted activity (∑β_w_) by the sum of the activity (∑β).


$$CoG=\frac{\Sigma \beta_{w}}{\Sigma \beta }$$

When comparing changes in the CoG for the hand or a finger, the CoG for each post-session was subtracted by the average CoG of the pre-sessions (e.g., 3m CoG – Pre. Avg CoG). A value greater than zero reflects the CoG moving more medially in the post session compared to the pre. A value less than zero reflects the post CoG being more lateral compared to the pre.

#### Finger selectivity maps

To visualize selectivity maps, estimates from the four finger-mapping scan runs for each session were averaged in a voxel wise manner using a fixed effects model with a cluster forming z-threshold of 3.1 and family-wise error corrected cluster significance threshold of *p* < 0.05. When visualizing the clusters, we stack them such that the smallest cluster is the highest overlay (e.g. the pinky) and the largest cluster is the underlay.

#### Representative control subject body-part maps

**Figure 5.**
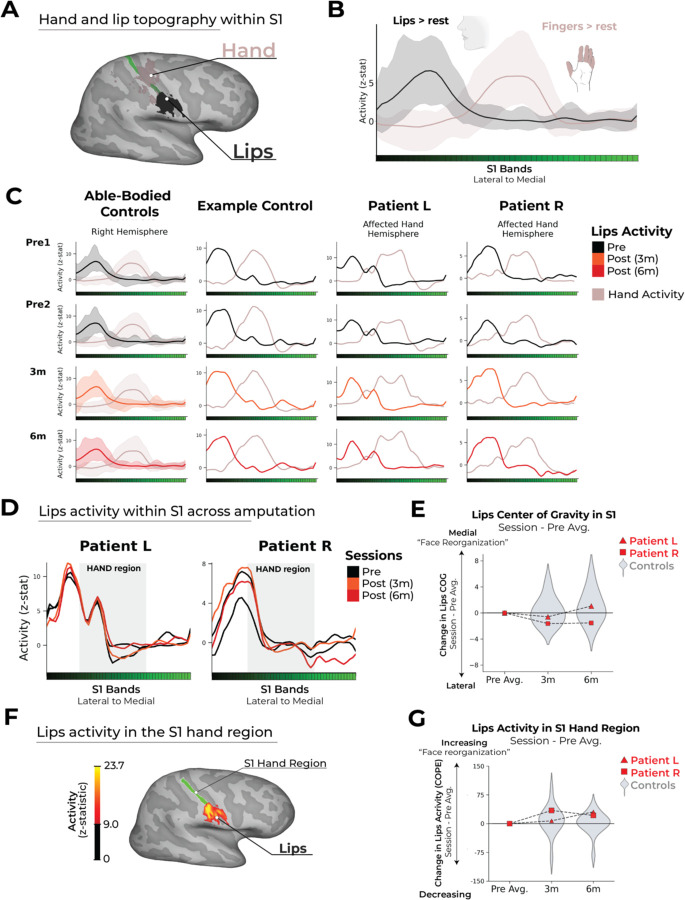
No evidence for large-scale reorganization after amputation. (A) Visualization of a representative control participant’s activity for the hand and lips (contrasted against feet; minimally thresholded at Z > 9) along with the S1 bands ROI outlined. (B) Control’s group activity (95% percentile interval) for each body part (versus baseline) projected onto the BA3b ROI. (C) Controls (95% percentile interval), an example control participant, and each patient’s affected/non-dominant hand and lip activity projected along the ROI over the four sessions (each row). (D) Patients’ longitudinal lips activity (versus rest) projected along the ROI over four sessions (indicated in the legend). (E) CoG shifts across amputation. Positive values reflect medial shifts (towards the hand). (F) Visualization of a representative control subject’s lip activity with activity scale and the hand ROI outlined. (G) Change in average lips activity within hand ROI over amputation. Increased lips activity within the S1 hand region post-amputation is indicated by higher values relative to pre-sessions. Patients inter-session differences were consistent with those observed in controls. All other annotations are the same as described in Figure 2.

To provide an example visualization of the activity for each of the body-parts (shown in Figure 5A), estimates from the four finger-mapping scan runs for each session were averaged in a voxel wise manner using a fixed effects model with a cluster forming z-threshold of 3.1 and family-wise error corrected cluster significance threshold of *p* < 0.05. We then visualized the z-statistic map for the contrast of lips > feet and all left fingers > feet on an inflated cortical surface and applied a threshold to each body-part (Z > 9).

#### Whole body topography across 49 bands of BA3b

Using the 49 bands of BA3b, we projected the neural activity for the 3 major body parts (hand, lips and feet; versus rest) for each hemisphere (contralateral to the hand being moved), session and participant. The average activity across all voxels within each band was averaged to extract a single value per band.

#### Lips activity in BA3b hand region

To test whether there is an increase in lip activity within the BA3b hand region, the average activity for all voxels (non-thresholded) in the ROI was computed for each session and each run. Activity was averaged across runs to compute a session estimate. When testing for a difference between the post and pre amputation sessions, the activity for the two pre-sessions was averaged for a pre avg. estimate. The activity in each post-amputation session (3m, 6m) was then subtracted to the activity of the pre avg.

### Multivoxel Pattern Analyses

We performed several multi-voxel pattern analyses that can be broadly categorized into two themes: intra-finger and inter-finger. In these measures, we were interested in capturing differences within a session and differences between sessions. For all of these analyses, we only included voxels within the BA3b hand region.

### Intra-finger

#### Pearson correlations

We first wanted to quantify changes in the pattern of activation for single fingers (intra-finger). We performed Pearson correlations on the beta-weights for each finger using data from (1) within the same session (i.e., between runs; Supp. Figure 3) or (2) runs from different sessions (Figure 3B–C). For within-session correlations, the beta-weights [in our instance, contrast of parameter estimates (COPE)] for each finger in the 4 scan runs were separated into partitions each with 2 runs. The activity within these 2 runs were averaged at every voxel. A Pearson correlation was then performed between the averaged activity in each of the splits. This method was performed for all 2-run unique combinations (3 total). All correlation coefficients were then averaged and plotted in Supp. Figure 3. Additionally, for the two patients, the minimum and maximum values from these within-session correlations for each finger pair were visualized on the between-session correlations as a grey shaded area to provide a noise-ceiling (Supp. Figure 3).

Next, we performed correlations between scan runs from different sessions. We used the same method, as the within-session, however we performed all unique 2-run combinations between-sessions (36 total combinations) and averaged these correlation coefficients to get a single value per finger. Between-session correlations were performed for all 6 unique session comparisons: Pre1 to Pre2, Pre1 to 3m, Pre1 to 6m, Pre2 to 3m, Pre2 to 6m, and 3m to 6m. For simplicity, we plotted Pre Avg. to 3m and Pre Avg. to 6m in Figure 3C.

### Inter-finger

We next wanted to quantify changes in the pattern of activation between finger pairs (inter-finger) using a decoding approach and cross-validated mahalanobis distances (Figure 4B). Both approaches capture slightly different aspects of the representational structure (71), which we elaborate on below.

For these two analyses, the beta-weights from the first-level GLM for each subject were extracted and spatially pre-whitened using a multivariate noise-normalization procedure [as described in (71)]. This was done using the residuals from the GLM, for each scan. We then used these noise-normalized beta-weights for the next analyses.

#### Decoding

First, we performed a decoding analysis. A strength of this approach is that it provides an estimate for chance performance (50%), i.e., *is the classification accuracy significantly greater than chance*. For the patients, the decoding approach can tell us whether a decoder trained on pre-amputated finger pairs can correctly decode the same information on a phantom hand.

We used a linear support vector machine classifier (scikit-learn v.1.2.2; sklearn.svm, LinearSVC). The default parameters were used for the classifier. Classification accuracy above chance (50%) denotes there is some amount of shared information between the train and test datasets.

We first quantified within-session decoding (i.e., between runs) for each finger pair. We trained the classifier on the noise-normalized beta-weights for each finger pair (10 total) for each 2-run combination and tested the classifier on the same finger pair activity in every other 2-run combination from the same session (3 in total). We performed both the forward and reverse comparisons, so 6 train/test splits in total. These classification accuracies were averaged for each finger pair across the train/test splits. All between and within-session classification accuracies are shown in Supp. Figure 5A–B.

Next, the train/test splits were performed using data from different sessions, such that the classifier was trained on each unique 2-run combination from one session and tested on all unique 2-run combinations in a separate session (36 combinations for each finger pair). We also performed the same classification approach in the reverse direction (72 total combinations) because the forward and reverse directions provide unique values. The accuracies for each finger pair for each 2-run combination for each train/test direction were then averaged. Between-session accuracies are shown in Figure 4C.

#### Cross-validated mahalanobis distances

Because our decoding analysis performed at ceiling (close to 100%), we also performed a representational similarity analysis using cross-validated mahalanobis distances. The strength of this approach is that it computes a distance measure (continuous) as opposed to a binary decoding measure. As such, it is arguably more sensitive for capturing the inter-finger representational structure. Larger distances reflect more dissimilar (distinct) activity patterns and smaller distances reflect more similar patterns.

We performed this analysis using either (1) data from within the same session to compute within-session cross-validated distances and (2) data from different sessions to compute between-session distances (our desired measure for representational stability over time). A distance cross-validated between sessions captures the stability of the information content.

We calculated the squared cross-validated Mahalanobis distance between activity patterns:

$$d^{2}\left(x_{y},x_{z}\right)={\left(x_{y}-x_{z}\right)}_{A}^{T}\Sigma^{-1}{\left(x_{y}-x_{z}\right)}_{B}$$

where ${\left(x_{y}-x_{z}\right)}_{A}$ corresponds to the difference between the activity patterns of conditions y (e.g., thumb) and z (e.g., index finger) in partition A, and Σ refers to the voxel-wise noise covariance matrix. We performed this procedure over all possible 2-run cross-validation folds and then averaged the resulting distances across folds. The number of cross-validation folds varied whether we performed the analysis on the data within a session (3 folds) or between-sessions (36 folds; please note that the cross-validated distance gives you the same distance value regardless of whether its assigned partition A or partition B). Within-session distances are shown in Supp. Figure 5D and between-session distances are shown in Figure 4E (and in entirety in Supp. Figure 5C).

#### Typicality

To quantify a measure that represents the degree of ‘normality’ of the hand representation, we computed a representational typicality measure (9). This was done by computing the Spearman’s rho correlation between the cross-validated mahalanobis finger-pair distances for each subject’s affected or non-dominant (left) hand and the mean distances of the non-dominant hand of the control subjects. When comparing a control subject to the control mean, the subject was left out from the estimation of the control mean distances.

### Statistical Analyses

All statistical analyses were performed using either python scripts utilizing scipy.stats and statsmodels.stats.multitest or JASP (0.17.2.1). Tests for normality were conducted using a Shapiro–Wilk test. For the majority of analyses, to test whether a patient was significantly different from the control group, we used Crawford and Howell’s method which provides a point estimate of the abnormality of the individual case’s distance from a control sample (72). For all Crawford tests, we provide the two-tailed p-values. When comparing estimates to 0 or chance decoding (50%), we used a one-sample t-test (two-tailed). When testing for a decrease in measures within-patients, we used a non-parametric Wilcoxon-Signed Ranked test. Additionally for the correlation analyses, Pearson correlations were used for the intra-finger multivoxel pattern analysis and Spearman correlations were used for the typicality analysis. To control for the multiple statistical tests (e.g., testing every finger pair) being performed for each analysis, we performed the standard FDR correction procedure also known as the Benjamini and Hochberg procedure (73). This correction was performed for all analyses where the total number of tests per patient was greater than 2. Correction for multiple comparisons was carried out for each patient separately.

## Supplementary Material

Supplement 1

Supplement 2

Supplement 3

## Figures and Tables

**Table 1.** Patient demographics. PLS = phantom limb sensation; PLP = phantom limb pain. Frequency scores: 1 – all the time, 2 – daily, 3 – weekly, 4 – several times per month, and 5 – once or less per month. Chronic pain/sensation values were calculated by dividing intensity by frequency. NA = not available/applicable.

	Patient L	Patient R
**Sex**	Female	Female
**Age**	26	57
**Handedness at birth**	Left-handed	Right-handed
**Cause of amputation**	Arteriovenous vascular malformation (AVM)	Sarcoma tumour
**Disability duration**	AVM progressed over a few years	Tumour slowly developing since 1995
**Amputated limb**	Left upper limb	Right upper limb
**Level of amputation**	Transhumeral	At elbow
**Amputation surgery**	Combination of targeted muscle reinnervation and regenerative peripheral nerve interfaces, see Supp. Fig. 8.	Traditional: sharply divided the major nerves and allowed to retract
**Phantom position and mobility**	Phantom hand positioned slightly above the elbow; only feels the hand, not the forearm; can move all phantom fingers (Figure 1B.	Phantom hand positioned upright towards chest; only feels the hand, not the forearm; can move all phantom fingers (Figure 1B).
**When did phantom sensations occur**	Immediately after amputation	Immediately after amputation
**PLS intensity** (100 max) (3m, 6m)	40, 60	90, 100
**PLS frequency** (3m, 6m)	Once a week, several times per month	All the time, all the time
**Chronic PLS** (100 max) (3m, 6m)	13.3, 15	90, 100
**PLP intensity** (Pre, 3m, 6m)	90, 20, 0	80, 50, 70
**PLP frequency** (Pre, 3m, 6m)	All the time, several times per month, once or less per month	All the time, daily, daily
**Chronic PLP** (Pre, 3m, 6m)	90, 5, 0	16, 25, 35
**Transient** (on the day) **pain** (100 max) (Pre, 3m, 6m)	50, 30, 0	80, 45, 50
**Pain Detect Score (% max possible score)** (Pre, 3m, 6m)	51%, 34%, 14%	68%, NA, 42%
**Pain Detect Pain Course**	Persistent pain with pain attacks (Same pre and 3m) Persistent pain with slight fluctuations (6m)	Persistent pain with pain attacks (Same pre and 6m)
**Upper Extremity Functional Index** (Pre, 3m, 6m) 100% = no impairment	45%, 21%, 32%	30%, NA, 11%
**Prosthesis Type**	None	Cosmetic
**Prosthesis Use** (6m)	NA	Occasional use: ~2 days a week, ~2 hours a day
