# Stable Cortical Body Maps Before and After Arm Amputation

**DOI:** 10.1101/2023.12.13.571314

**Published:** 2025-02-04

**Authors:** Hunter R. Schone, Roni O. Maimon Mor, Mathew Kollamkulam, Malgorzata A. Szymanska, Craig Gerrand, Alexander Woollard, Norbert V. Kang, Chris I. Baker, Tamar R. Makin

**Affiliations:** 1 Institute of Cognitive Neuroscience, University College London, London, UK; 2 Laboratory of Brain & Cognition, National Institutes of Mental Health, National Institutes of Health, Bethesda, Maryland, USA; 3 Rehab Neural Engineering Labs, University of Pittsburgh, Pittsburgh, PA, USA; 4 Department of Physical Medicine and Rehabilitation, University of Pittsburgh, Pittsburgh, PA, USA; 5 Department of Experimental Psychology, University College London, London, UK; 6 UCL Institute of Ophthalmology, University College London, London, UK; 7 Department of Experimental Psychology, University of Oxford, Oxford, UK; 8 MRC Cognition and Brain Sciences Unit, University of Cambridge, Cambridge, UK; 9 Department of Orthopaedic Oncology, Royal National Orthopaedic Hospital NHS Trust, Stanmore, Middlesex, UK; 10 Plastic Surgery Department, Royal Free Hospital NHS Trust, London, UK; 11 Wellcome Centre for Human Neuroimaging, UCL Institute of Neurology, London, UK

## Abstract

The adult brain’s capacity for cortical reorganization remains debated. Using longitudinal neuroimaging in three adults, followed up to five years before and after arm amputation, we compared cortical activity elicited by movement of the hand (pre-amputation) versus phantom hand (post-amputation) and lips (pre/post-amputation). We observed stable representations of both hand and lips. By directly quantifying activity changes across amputation, we overturn decades of animal and human research, demonstrating amputation does not trigger large-scale cortical reorganization.

What happens to the brain’s map of the body when a part of the body is removed? Over the last five decades, this question has captivated neuroscientists and clinicians, driving research into the brain’s capacity to reorganize itself. Primary somatosensory cortex (S1), known for its highly detailed body map, has historically been the definitive region for studying cortical reorganization^1,2^. For example, foundational research in monkeys reported that, following an amputation or deafferentation, the affected region within the S1 body map suddenly responds to inputs from cortically-neighboring body-parts (e.g., face)^3,4^. Additional neuroimaging studies in human amputees supported the theory that amputation of an arm triggers large-scale cortical reorganization of the S1 body map^5–7^, with a dramatic redistribution of cortical resources, hijacking the deprived territory^1^.

Recent studies have challenged this view by harnessing human amputees’ reports of experiencing vivid sensations of the missing (phantom) limb. First, human neuroimaging studies have demonstrated that voluntary movements of phantom fingers engage neural patterns resembling those of able-bodied individuals^8–10^. Second, phantom sensations are evoked by cortical^11^ or peripheral^12,13^ nerve stimulation, suggesting an intact neural representation of the amputated limb, despite its physical absence. Third, neuroimaging studies using both tactile stimulation and movement paradigms reported no changes in face or lip activity within the deprived cortex of adult amputee participants compared to able-bodied controls^14,15^, (though remapping observed in children)^16^.

This debate—whether or not amputation triggers large-scale reorganization—remains unresolved^17,18^, with some suggesting the two views are not conceptually exclusive – preservation and reorganization can co-exist^5,19,20^. However, a fundamental issue with the evidence on both sides of this debate is a methodological reliance on cross-sectional designs (i.e., comparing between participants). While offering valuable proofs of concept, these studies cannot determine whether the maps of the phantom hand or face are truly preserved or changed, relative to their pre-amputation state. To directly track the evolution of cortical representations before and after amputation, we implemented a longitudinal fMRI approach to track the cortical representations of the hand and face (lips) in three adult participants up to 5 years after arm amputation (Video 1), compared with able-bodied control participants (Figure 1A). Avoiding the confounding effects of cross-sectional designs^21^, we directly quantify the impact of arm amputation on S1 (re)organization.

We studied three adult participants (case-studies: P1, P2, P3) undergoing arm amputation (demographics in Supp. Table 1) across 4–5 timepoints, and 16 able-bodied controls at 4 timepoints over 6 months (Figure 1A). Pre-amputation, all participants could move all fingers, to varying ranges (Supp. Figure 1 and Supp. Video 1). Post-amputation, all participants reported vivid phantom limb sensations (Figure 1B), including volitional phantom fingers movement (Supp. Table 1 and Supp. Figure 1). Motor control over the phantom hand was further confirmed by residual limb muscle contractions during phantom movements (Supp. Video 1), and selective activation in primary sensorimotor cortex for attempted, but not imagined, phantom movements (Figure 1C). The critical question is to what degree S1 phantom activity reflects the pre-existing hand.

During scanning, participants performed visually-cued movements involving tapping individual fingers, pursing lips, and flexing toes. Case-study participants demonstrated strikingly consistent hand and lip cortical maps before and after amputation (Figure 1D). Projecting hand and individual fingers activity profiles across S1 revealed stable activity before and after amputation, with phantom activity resembling the pre-amputation amplitude and spatial activity spread (Figure 2A). A center of gravity (CoG) analysis of these profiles revealed spatially consistent hand and individual finger activity in our case-studies, with similar pre- to post-session differences over 6 months as controls (six Crawford t-tests per participant: P1: 0.14≤*p*_*uncorr*_≤0.58; P2: 0.06≤*p*_*uncorr*_≤0.81; P3: 0.10≤*p*_*uncorr*_≤0.91). Notably, this stability cannot be attributed to a pre-existing baseline difference, as hand activity pre-amputation was normal relative to controls (Supp. Figure 2A). Similar pre-post stability was observed in motor cortex (M1; Supp. Figure 3A) and for the intact (unaffected) hand (Supp. Figure 4A).

Next, we investigated S1 finger representation stability in greater detail using a multi-voxel pattern analysis (Figure 2B; Methods). Multi-voxel activity patterns for the pre-amputated versus phantom fingers were significantly correlated at 6 months [five Pearson correlations per participant; P1: 0.68≤ *r*≤ .90, *p*_*uncorr*_*<*0.001; P2: 0.80≤*r*≤.85, *p*_*uncorr*_*<*0.001; P3: 0.88≤*r*≤.91, *p*_*uncorr*_*<*0.001]. Correlation coefficients at 6 months fell within the typical distribution seen in controls (see Supp. Figure 5 and Supp. Table 2 for control values). Similar stability was observed in M1 (Supp. Figure 3) and for the intact hand (Supp. Figure 5). Combined, this confirmed that activity was largely stable before and after amputation at the single voxel level.

We next considered finger selectivity, i.e. activity profiles for each finger versus other fingers. Qualitative finger mapping revealed preserved somatotopy before and after amputation (Figure 2C). We applied a multivoxel pattern analysis using a linear support vector machine classifier (Figure 2D) to explore whether a pre-amputation-trained classifier can decode phantom finger movements (and vice versa). This analysis revealed significantly above chance classification for all case-study participants across all post-amputation sessions [Figure 2D; 2–3 one-sample t-tests per participant: P1 (Pre/1.5y): 90%; t(9)=10.5, *p*_*uncorr*_*<*0.001; P2 (Pre/5y): 67%; t(9)=4.85, *p*_*uncorr*_*<*0.001; P3 (Pre/6m): 89%; t(9)=11.0, *p*_*uncorr*_*<*0.001], with similar evidence in M1 (Supp. Figure 3).

We next investigated whether amputation reduces finger selective information, as suggested by previous cross-sectional studies^22^. Assessing for abnormalities in the pre-amputation data, we noted that 1 of the case-study participants, P2, exhibited lower classification for the pre-amputated hand relative to controls (Supp. Figure 2), likely due to P2’s impaired motor control pre-amputation (Supp. Video 1). Our key question remains whether this information degrades further following amputation. When comparing selectivity differences over 6 months relative to controls, none of the case-study participants showed significant reductions in average finger selectivity (Crawford t-test: P1: t(15)=−0.34, *p*=0.73; P2: t(15)=−0.24, *p*=0.80; P3: t(15)=−1.0, *p*=0.33; Supp. Figure 6C). While finger selectivity was reduced at P2 and P3’s final scan relative to their baseline (Figure 2D; 3 Wilcoxon tests per participant: P1 (1.5y): W=3.0, *p*_*uncorr*_=0.11; P2 (5y): W=2.0, *p*_*uncorr*_=0.005; P3 (6m): W=1.0, *p*_*uncorr*_=0.01), these reductions could be attributed to the much greater longitudinal variability between training and testing classifier samples^23^. Therefore, any reductions in finger selectivity could not be directly attributed to the amputation.

We also performed a complementary representational similarity analysis (RSA) using Mahalanobis distances (a continuous measure of finger selectivity), cross-validated across sessions. Similar to the decoding, RSA confirmed finger-selective information was significantly consistent across amputation for all case-study participants at all post-amputation timepoints (2–3 one-sample t-tests per participant: *p*_*uncorr*_<0.0001; Supp. Figure 6), with similar evidence in M1 (Supp. Figure 3C). We noted a few temporary, idiosyncratic (uncorrected) instances of reduced finger selectivity, relative to controls (Supp Figure 6). Using the RSA distances, we also tested the typicality of the inter-finger representational structure, an additional feature of hand representation. Correlating each participant’s inter-finger pattern to a canonical pattern revealed no deterioration in typicality scores 6-months post-amputation, compared to controls, with P3 even showing higher typicality than the average control (Crawford t-test: P1: t(15)=−0.9, *p*=0.38; P2: t(15)=−0.9, *p*=0.38; P3: t(15) = −3.5, *p*=0.003; Supp. Figure 6). Therefore, despite idiosyncratic reductions in finger selectivity, the representational structure was preserved post-amputation.

Finally, we examined changes in the lip representation, previously implicated with reorganization following arm amputation^4,7^. Projecting hand and lip univariate activity onto the S1 segments revealed no evidence of lip activity shifting into the hand region post-amputation (Figure 3A). All case-study participants showed typical longitudinal variability at their 6 months scan, relative to controls, for lip CoG [Figure 3B; Crawford t-test: P1: t(15)=0.25, *p*=0.80; P2: t(15)=−0.89, *p*=0.38; P3: t(15)=−0.9, *p*=0.37]. Further, lip activity in the S1 hand region at the final scan was typical [Figure 3C; P1 (1.5y): t(15)=0.8, *p*=0.20; P2 (5y): t(15)=−0.5, *p*=0.71; P3 (6m): t(15)=1.2, *p*=0.10]. Also, when visualizing the lip map boundaries within S1 for all sessions, using a common minimum threshold, there was no evidence for an extension of the lip map (Figure 3D). Examining multivariate lip representational content, P2 showed an increased lip-to-thumb multivariate distance at their 6 months scan, relative to controls [Figure 3E; Crawford t-test: P1: t(15)=0.69, *p*=0.25; P2: t(15)=3.1, *p*=0.003; P3: t(15)=.74, *p*=0.23; intact hand and feet data included in Supp. Figure 7] However, it returned to the typical range of controls when assessed at their 5-year timepoint. Similar stability was found in M1 (Supp. Figure 3), and the unaffected hemisphere (Supp. Figure 4). These results demonstrate that amputation does not affect lip topography or representational content in S1.

To complement our longitudinal findings, we compared our case studies to a cohort of 26 chronic upper-limb amputee participants, on average 23.5 years post-amputation (Figure 3F; individual hand and lip cortical maps in Supp. Figure 8). Our case-studies’ topographical features were comparable to chronic amputees for both the phantom hand [Crawford t-test: P1 (1.5y): t(15)=0.28, *p*=0.77; P2 (5y): t(15)=0.29, *p*=0.77;*p*=0.77; P3 (6m): t(15)=0.28, *p*=0.22; *p*=0.82] and lips [P1 (1.5y): t(15)=0.53, *p*=0.59; P2 (5y): t(15)=0.01, *p*=0.98; P3 (6m): t(15)=0.37, *p*=0.71]. Average lip activity within the S1 hand region was slightly (though not significantly) higher for a few of our case-studies relative to chronic amputees (Crawford t-test: P1 (1.5y): t(15)=1.6, *p*=0.10; P2 (5y): t(15)=0.24, *p*=0.81; P3 (6m): t(15)=1.8, *p*=0.065), reflecting that lip activity does not steadily increase in the years after amputation. Collectively, these results provide long-term evidence for the stability of hand and lip representations despite amputation.

Beyond the stability of the lip representation across amputation, our findings reveal highly consistent hand activity despite amputation. This unchanged hand representation challenges the foundational assumption that S1 activity is primarily tied to peripheral inputs, suggesting that S1 is not a passive relay of peripheral input, but an active supporter of a resilient ‘model’ of the body—even after amputation. We therefore conclude that, in the adult brain, S1 representation can be maintained by top-down (e.g. efferent) inputs. This interpretation sheds new light on previous studies showing similar S1 topographical patterns activated by touch^24^, executed movement^25^ and planned movement^26^.

Due to the limitations of non-human models that cannot communicate phantom sensations, it is not surprising that the persistent representation of a body part, despite amputation, has been neglected from previous studies. Without access to this subjective dimension, researchers may have missed the profound resilience of cortical representations. Instead, previous studies determined S1 topography by applying a ‘winner takes all’ strategy –– probing responses to remaining body parts and noting the most responsive body part in the input-deprived cortex^3,4^. Ignoring phantom representations in these analyses leads to severe biases in the interpretation of the area’s inputs (as demonstrated in Supp. Figure 10). Combined with cross-sectional designs, this has incorrectly led to the impression of large-scale reorganization of the lip representation following amputation. Our longitudinal approach reveals no signs of reorganization in S1—not even subtle upregulation from homeostasis—further reinforcing the notion that S1 is not governed by deprivation-driven plasticity.

For brain-computer interfaces, our findings demonstrate a highly detailed and stable representation of the amputated limb for long-term applications^27^. For phantom limb pain treatments, our study indicates that targeted muscle reinnervation and regenerative peripheral nerve interfaces do not ‘reverse’ reorganization or alter the cortical hand representation^22,28^. Finally, our findings affirm the unaltered nature of adult sensory body maps following amputation, suggesting Hebbian and homeostatic deprivation-driven plasticity is even more marginal than considered by even the field’s strongest opponents of large-scale reorganization^17,29^.

## Supplementary Material

Supplement 1

Supplement 2

Supplement 3

## Figures and Tables

**Figure 1.**
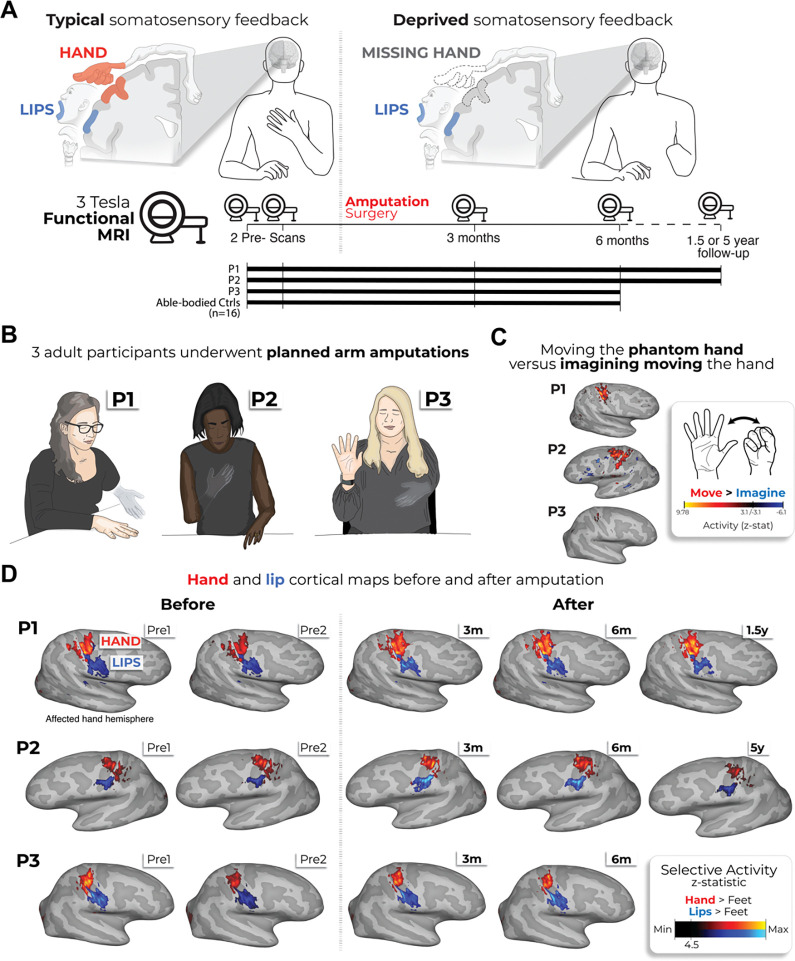
Longitudinal investigation of participants with planned arm amputations. **(A)** Experimental timeline. Pre- and post-amputation scans were conducted across 4–5 time points: twice before, and at 3 months, 6 months and 1.5 (P1) / 5 years (P2) after amputation. **(B)** Illustration depicting the 3 participants 6m post-amputation, including their subjective description of their phantom limb position. **(C)** Phantom movements are not imaginary. Univariate activity (z-scored) contrast map displaying participant’s attempts to open and close the phantom hand vs. imagining movement, 6 months post-amputation. **(D)** Participant’s hand (red) and lip (blue) cortical activation maps (contrasted against feet movements) within the affected hand hemisphere across 4–5 sessions. All maps were minimally thresholded at 33% the maximum z-statistic and used a common color scale (participant’s maximum Z-statistic > 4.5).

**Figure 2.**
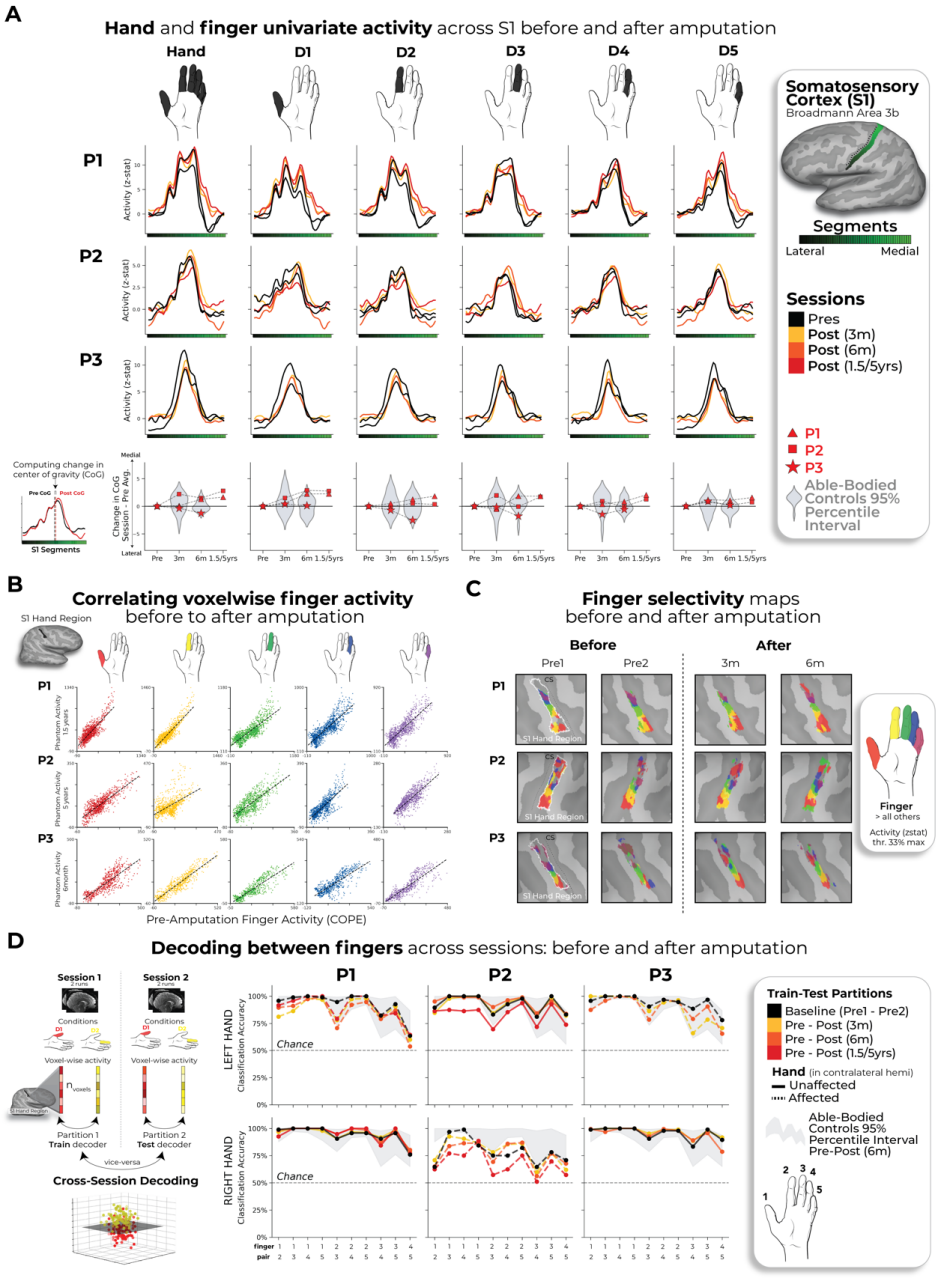
Stable hand representation within the affected hemisphere despite amputation. **(A)** Longitudinal hand and individual finger activity (versus rest) projected across the S1 (BA3b) region of interest (ROI) segmented into 49 segments of similar height. Affected hand’s activity over 5 sessions (indicated in the legend) for each of the case-study participants that underwent an amputation; bottom row shows finger CoG shifts before and after amputation. Black lines reflect pre-amputation activity, orange/red lines post-amputation. Case-study participants’ CoG shifts (red) for the hand and individual fingers fell within the distribution of controls (grey; 12–18 comparisons per participant; Crawford t-tests: P1 (6m): 0.14≤ p_uncorr_≤ 0.58; P2 (6m): 0.06≤p_uncorr_≤0.81; P3 (6m): 0.10≤p_uncorr_≤0.91). Values indicate group means ± standard error. Positive values indicate medial shifts (toward feet), negative values lateral (toward lips) in S1. Control data shown as gray violin plots. P1 data shown as a red triangle. P2 data shown as a red square. P3 data shown as a red star. For simplicity, the control values are all for the left (non-dominant) hand. **(B)** Pre-post amputation single-finger multi-voxel correlation: For each finger of the case-study participants, voxel-wise activity correlations before and at the final scan after amputation are shown. All other correlations are comprehensively reported in Supp Figure 5. All participant’s pre-to-post correlations were significant (5 Pearson correlations per participant; P1 (6m): 0.68≤ r≤ .90, p_uncorr_<0.001; P2 (6m): 0.80≤r≤.85, p_uncorr_<0.001; P3 (6m): 0.88≤r≤.91, p_uncorr_<0.001). **(C)** Finger selectivity maps before and after amputation**.** Each contrast map reflects the activity for each finger (versus all others), masked to the hand ROI. Each mask was minimally thresholded at 33% the maximum z-statistic. Color codes indicated on the right. To capture the multi-finger activity at a single voxel, a 70% opacity filter was applied to all fingers. **(D) Left** - Graphic illustration of multivoxel analyses using a linear SVM decoder. **Right** – Longitudinal classifier performance. Line colors denote train-test/cross validation session pairs, respectively as indicated in the legend. The gray shaded area reflects able-bodied control’s Pre – Post (6m) data (95% percentile interval). Training the classifier on the pre-amputation data and testing it on the post-amputation data (and vice versus) revealed significantly above chance classification accuracies for all case-study participants at all post-amputation sessions (one-sample t-test: P1: Pre/1.5y: 89%; p<0.001; P2: Pre/5y: 67%; p<0.001; P3: Pre/6m: 88%; p<0.001). All other annotations are depicted in Figure 1.

**Figure 3.**
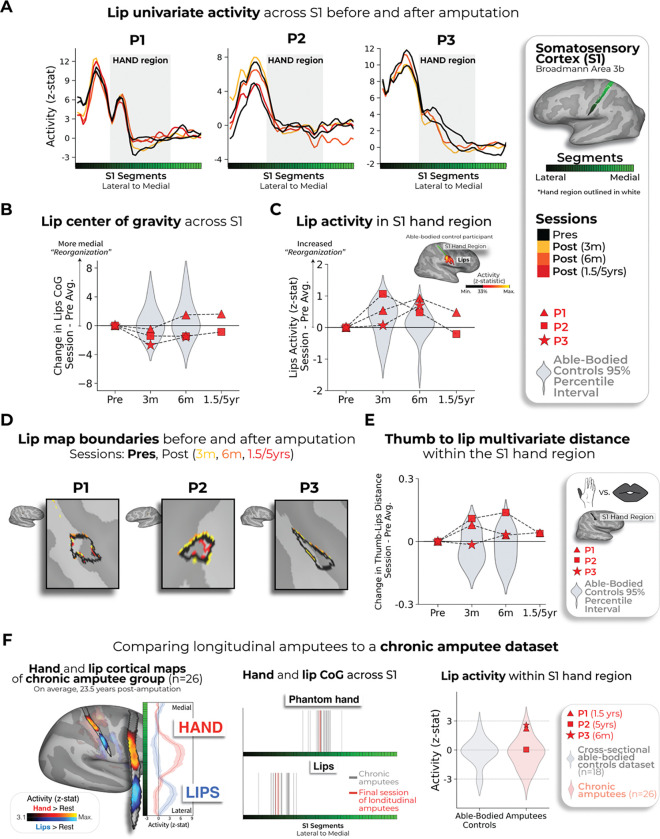
No evidence for lip reorganization after amputation. **(A)** Each case-study participant’s lip activity (versus rest) for their sessions projected across the S1 ROI. Black lines reflect pre-amputation activity, yellow (3m), orange (6m) and red (1.5/5y) lines post-amputation. Grey region depicts approximated coverage of the hand portion within S1. **(B)** All case-study participants showed typical longitudinal variability at their 6 months scan, relative to controls, for lip CoG. Positive values reflect medial shifts (towards the hand). **(C)** All case-study participants showed typical lip activity in the S1 hand region at the final scan. Right corner of panel depicts representative control participant’s activity for the hand and lips (versus feet; minimally thresholded at 33% the max. z-statistic). **(D)** All case-study participants exhibited no expansions of the lip map boundaries towards the hand region. Maps masked to the S1 ROI and minimally thresholded (Z > 4.5). **(E)** All case-study participants showed stable thumb-to-lip multivariate Mahalanobis distances cross-validated at their final scan, relative to controls. (F) Comparing the case-study participants to a chronic amputee dataset (n=26). **Left** – chronic amputee’s group-level cortical activation maps of the phantom hand and lips (versus rest) projected onto a single hemisphere (minimally thresholded at Z > 3.1). Opacity applied to activity outside the S1 ROI. Group univariate activity plotted as a line (group mean ± standard error) for the phantom hand (red) and lips (blue) across the S1 ROI. **Middle** – All case-study participants, comparable to chronic amputees, showed a typical center of gravity for both the phantom hand (top row) and lips (bottom row). **Right** – All case-study participants exhibited typical lip activity within the S1 hand region during their final session consistent with chronic amputees. The magnitude of lip activity (95% percentile interval) within the S1 hand region for a secondary able-bodied control group (n=18; shown in grey). Chronic amputees shown in pink and the case-study participants last session data shown in red. All other annotations are the same as described in Figure 2.

## Data Availability

Code and data used in the study will be made available following peer-reviewed publication.
