# Supplementary material for: Stable Cortical Body Maps Before and After Arm Amputation
Source: bioRxiv. 2023 Dec 14:2023.12.13.571314. Preprint. [Version 1] doi: 10.1101/2023.12.13.571314 (PMC10760201; doi:10.1101/2023.12.13.571314)
Supplement: Supplementary file 1 [file NIHPP2023.12.13.571314v1-supplement-3.pdf]

bioRxiv preprint doi: https://doi.org/10.1101/2023.12.13.571314; this version posted December 14, 2023. The copyright holder for this preprint (which was not certified by peer review) is the author/funder. This article is a US Government work. It is not subject to copyright under 17 USC 105 and is also made available for use under a CC0 license.

Supplementary Results

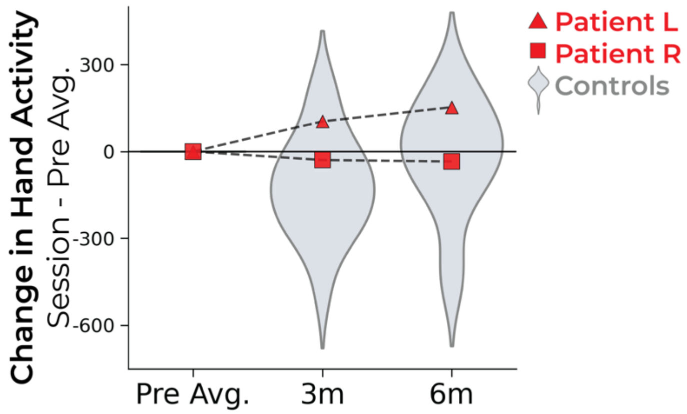

Supplementary Figure 1. Change in the magnitude of hand activity within the hand region of S1 across sessions. *Hand activity was computed as the average activity across all voxels within the hand region of S1. The data reflects the patient's amputated hand and the able-bodied controls left hand. All other annotations are the same as described in Figure 2.*

bioRxiv preprint doi: https://doi.org/10.1101/2023.12.13.571314; this version posted December 14, 2023. The copyright holder for this preprint (which was not certified by peer review) is the author/funder. This article is a US Government work. It is not subject to copyright under 17 USC 105 and is also made available for use under a CC0 license.

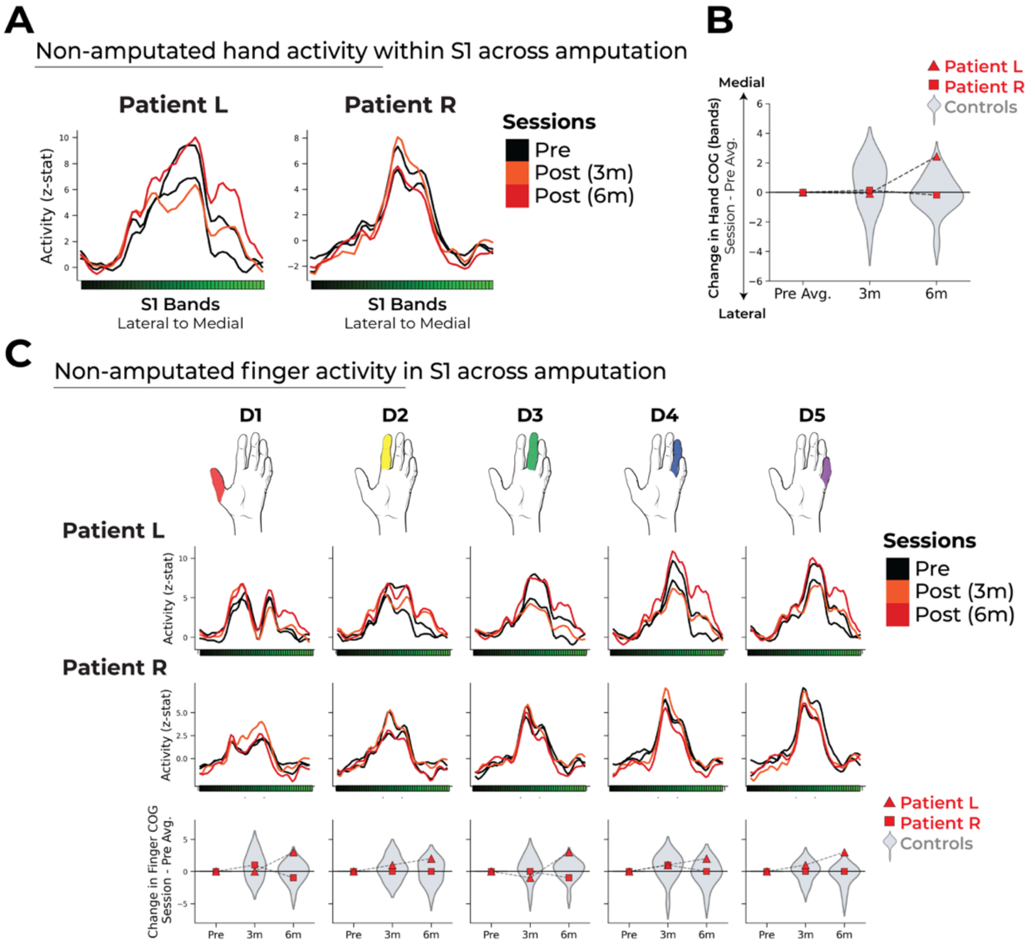

Supplementary Figure 2. Hand and finger topography of the intact (non-amputated) hand in the non-affected hemisphere across amputation. *All other annotations are the same as described in Figure 2.*

bioRxiv preprint doi: https://doi.org/10.1101/2023.12.13.571314; this version posted December 14, 2023. The copyright holder for this preprint (which was not certified by peer review) is the author/funder. This article is a US Government work. It is not subject to copyright under 17 USC 105 and is also made available for use under a CC0 license.

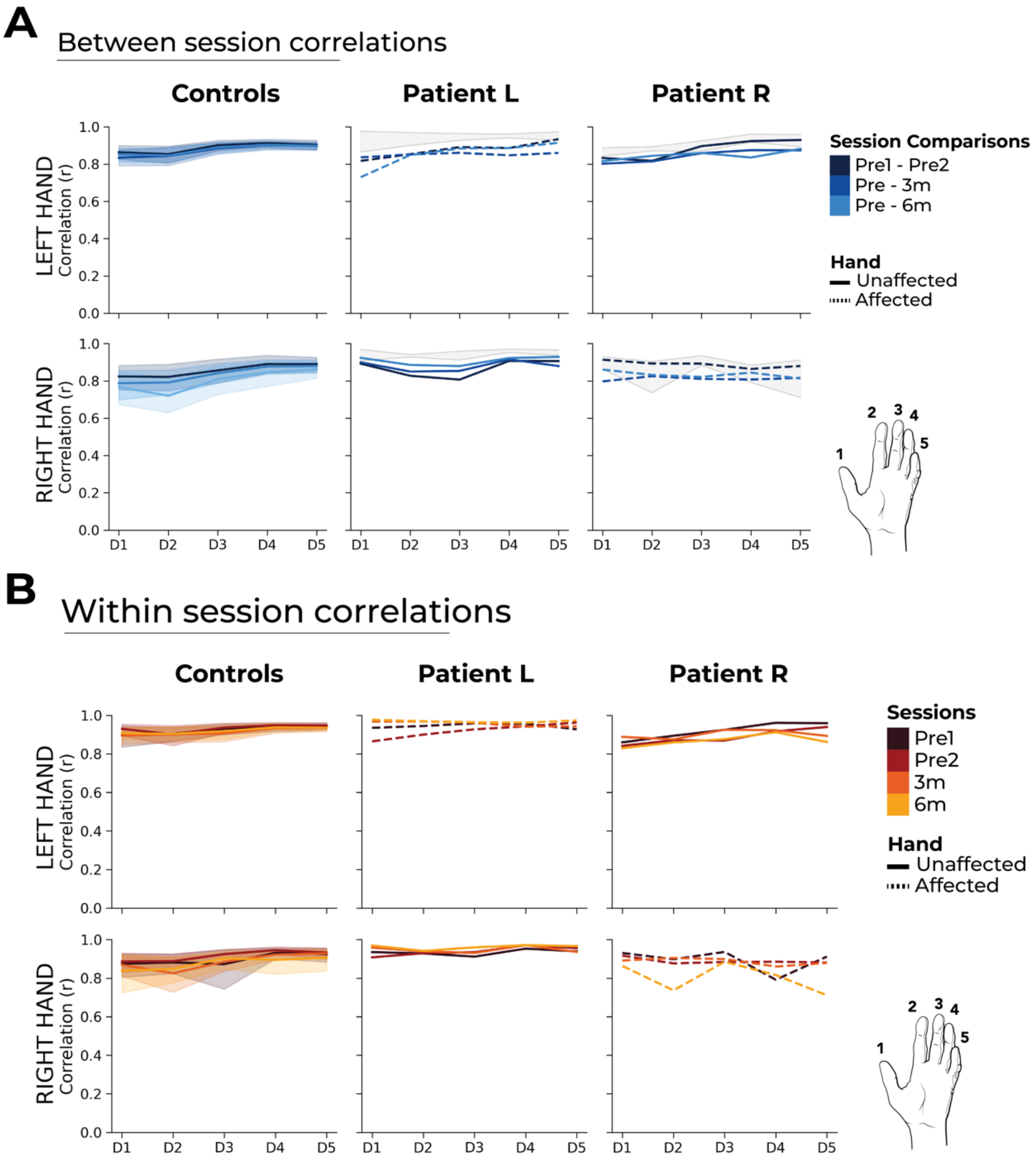

Supplementary Figure 3. Between and within-session intra-finger correlations. *The correlations are performed on finger activity within the hand ROI from different runs of (A) different sessions or (B) the same session (see Methods). For both patients, the minimum and maximum within-session intra-finger correlation values in panel B are depicted as noise-ceilings in the between-session data plotted in panel A. All other annotations are the same as described in Figure 3.*

bioRxiv preprint doi: https://doi.org/10.1101/2023.12.13.571314; this version posted December 14, 2023. The copyright holder for this preprint (which was not certified by peer review) is the author/funder. This article is a US Government work. It is not subject to copyright under 17 USC 105 and is also made available for use under a CC0 license.

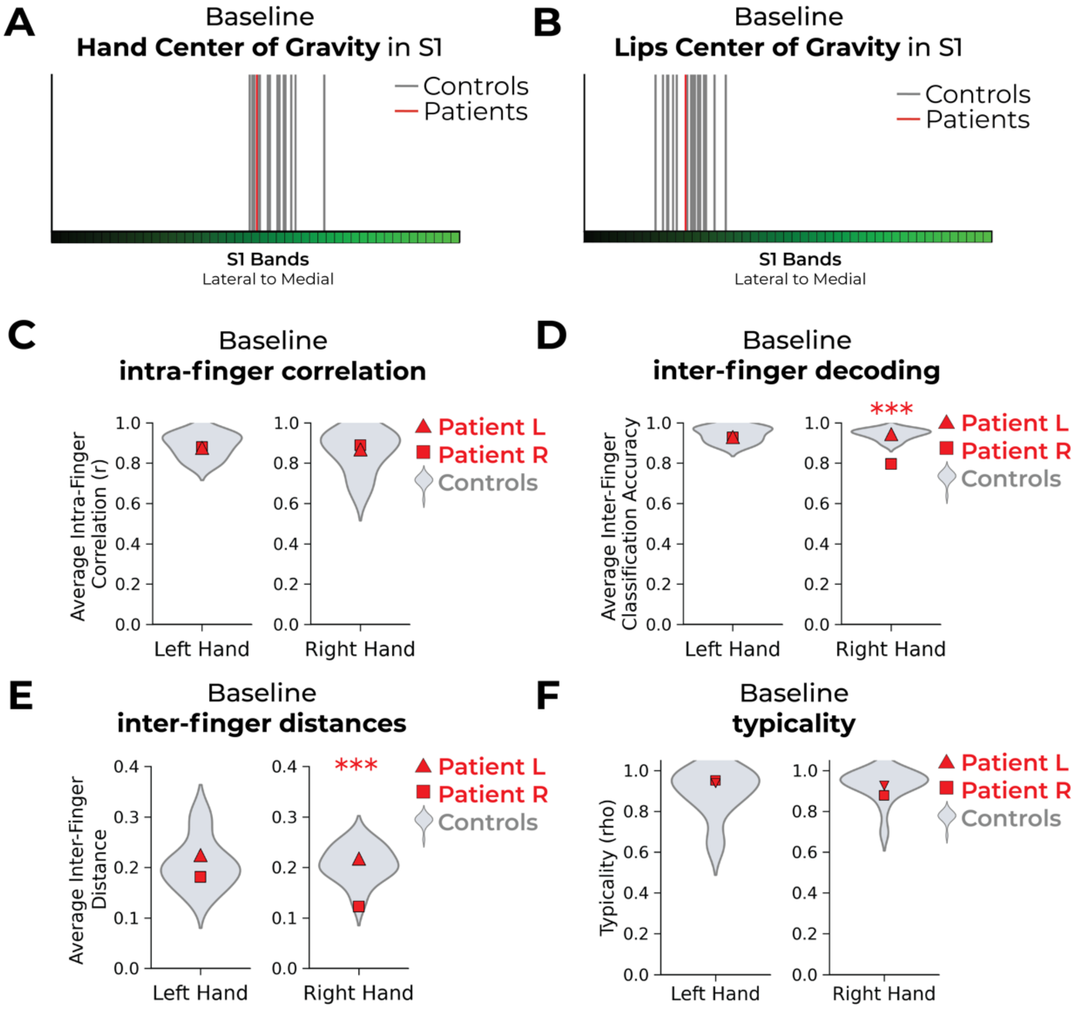

Supplementary Figure 4. Baseline measures for patients versus controls. *Patients showed similar responses to able-bodied controls in the baseline (pre-amputation) center of gravity for the (A) hand and (B) lips in S1. (C) Both patients had similar average intra-finger correlations between the two pre-sessions as controls. For baseline average inter-finger (D) classification accuracy and (E) distances, Patient L showed no baseline deficits, however Patient R exhibited lower values for their affected hand only, relative to controls. (F) Both patients had similar hand typicality between the two pre-sessions as controls. All other annotations the same as described in Figure 2.*

bioRxiv preprint doi: https://doi.org/10.1101/2023.12.13.571314; this version posted December 14, 2023. The copyright holder for this preprint (which was not certified by peer review) is the author/funder. This article is a US Government work. It is not subject to copyright under 17 USC 105 and is also made available for use under a CC0 license.

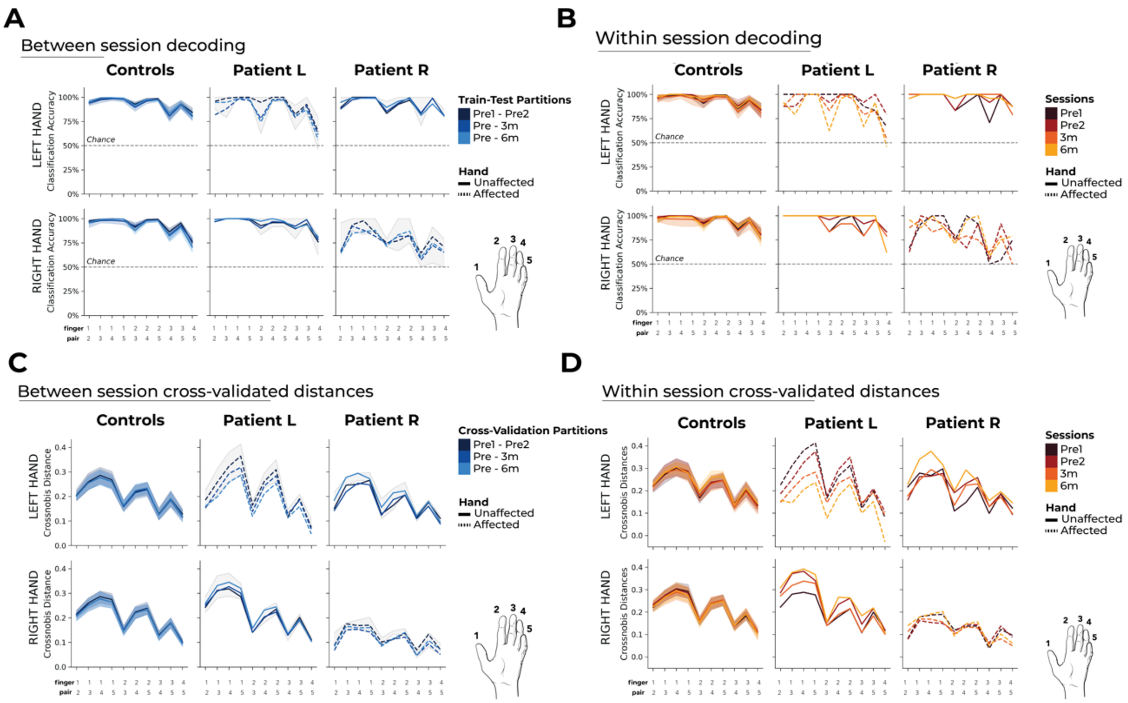

Supplementary Figure 5. Between and within-session inter-finger representational content. *Between- and within-session values for each finger pair in the contralateral hand ROI are shown for both (A-B) classification accuracies (C-D) and cross-nobis distances. Between session reflects the data coming from different scans from different sessions. Within session reflects data from different scans from the same session (see Methods). Line colours denote train-test/cross validation session pairs, respectively as indicated in the legend. For both patients, the minimum and maximum within-session inter-finger distances/accuracies are depicted as noise-ceilings in the between-session data. All other annotations are the same as described in Figure 2.*

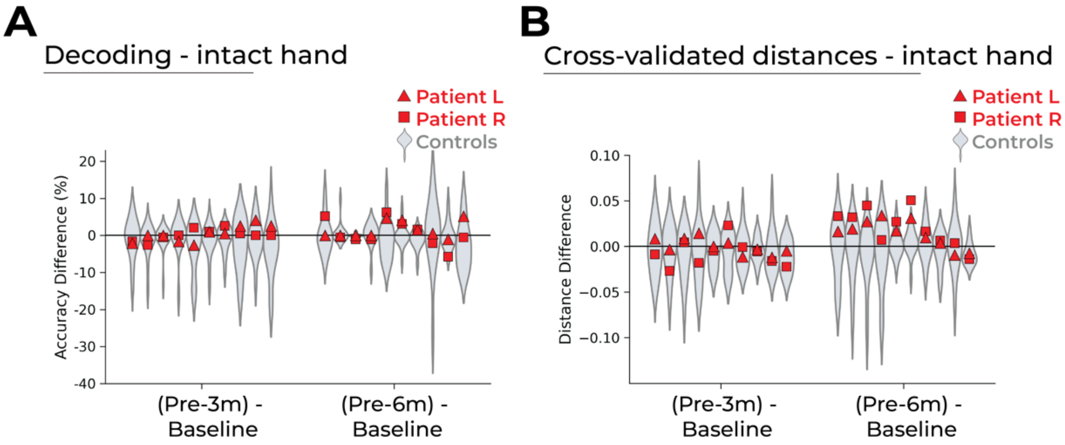

Supplementary Figure 6. Unaffected (intact) hand between-session differences in inter-finger values*. Difference values are depicted for the (A)*

bioRxiv preprint doi: https://doi.org/10.1101/2023.12.13.571314; this version posted December 14, 2023. The copyright holder for this preprint (which was not certified by peer review) is the author/funder. This article is a US Government work. It is not subject to copyright under 17 USC 105 and is also made available for use under a CC0 license.

decoding accuracies and (B) cross-validated distances. All annotations are the same as those in Figure 4D,F. For simplicity, the control values are all for the left-hand, same as depicted in Figure 4.

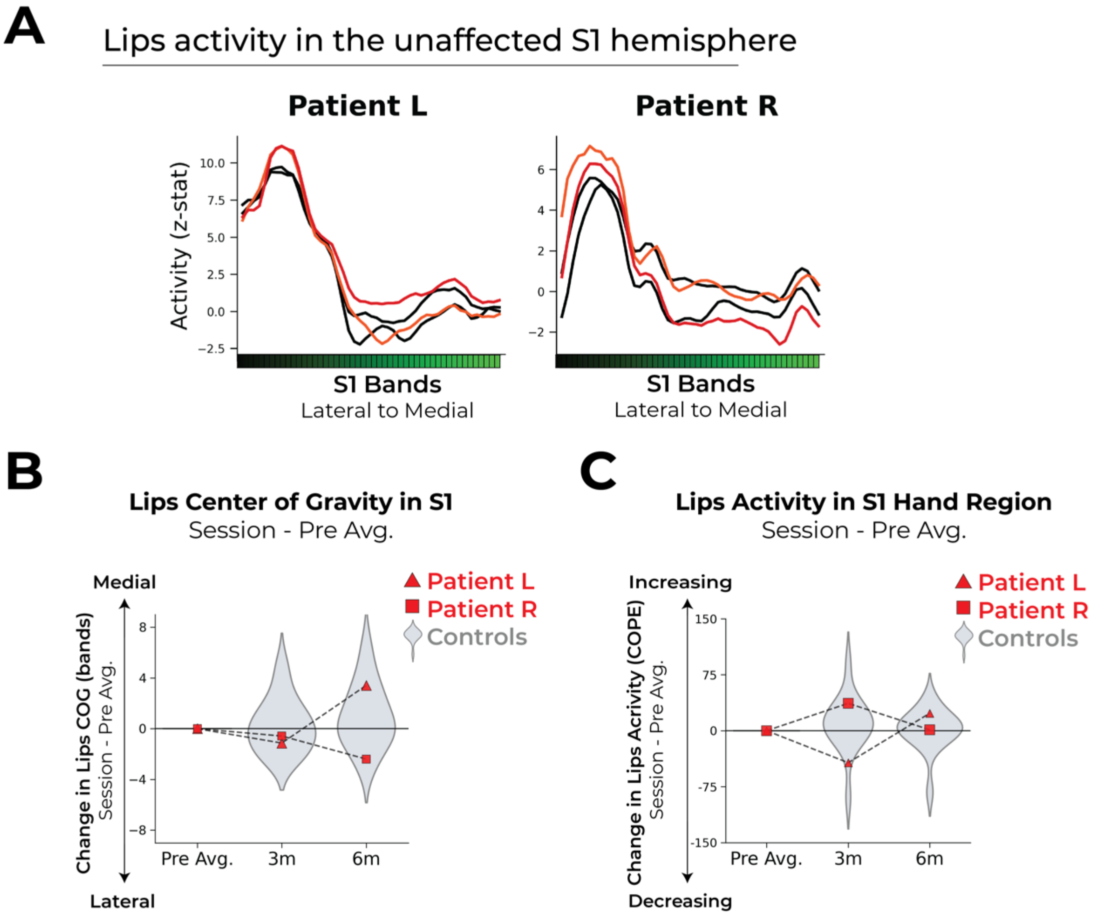

Supplementary Figure 7. Lip topography in the unaffected hemisphere across amputation. *All other annotations are the same as described in Figure 5.*

bioRxiv preprint doi: https://doi.org/10.1101/2023.12.13.571314; this version posted December 14, 2023. The copyright holder for this preprint (which was not certified by peer review) is the author/funder. This article is a US Government work. It is not subject to copyright under 17 USC 105 and is also made available for use under a CC0 license.

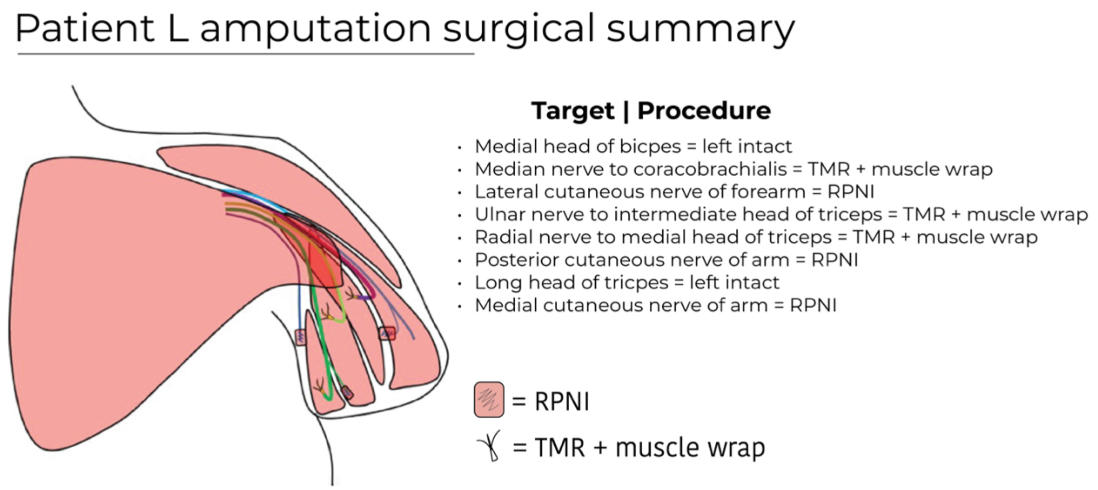

Supplementary Figure 8. Summary of Patient L's amputation procedure. *An illustration depicting the unique amputation surgery of Patient L's left arm, as well as summary of the procedures performed for each respective nerve. TMR=targeted muscle reinnervation; RPNI=regenerative peripheral nerve.*

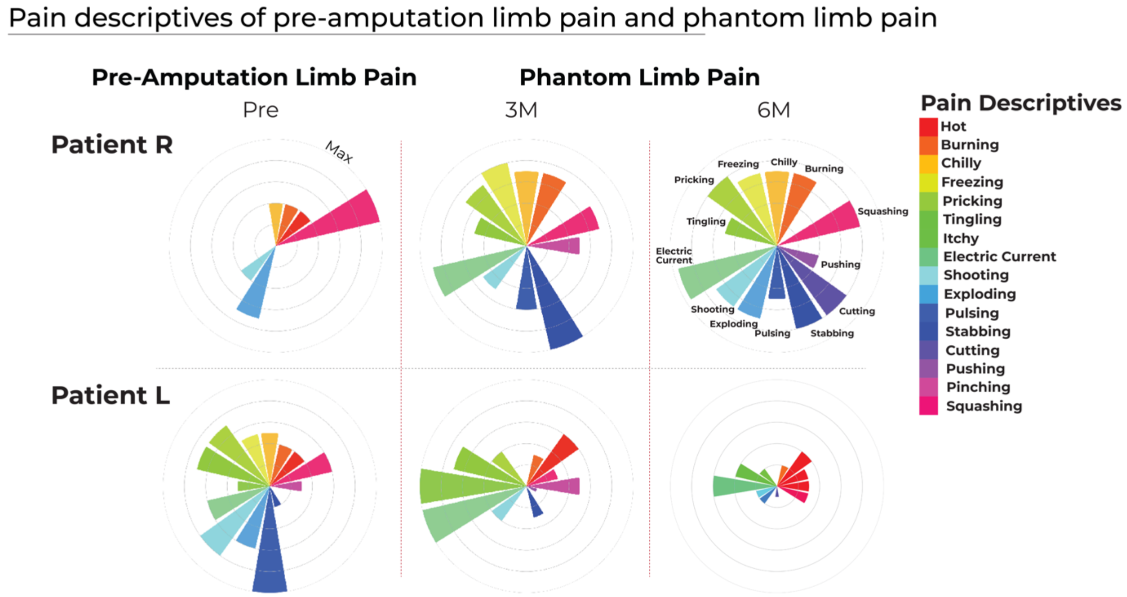

Supplementary Figure 9. Longitudinal characterization of pre-amputated limb pain and phantom limb pain. *Before and after amputation, both patients reported intensity values for each pain descriptive word, broadly categorized into sensations that are mechanical, temperature-related and other. For each word, participants were asked to describe the intensity between 0 (non-existing) to 100 (excruciating pain) as it relates to that particular word. A value of 100 (Max) is the largest radii on the polar plot. 3M=3months post-amputation; 6M=6months post-amputation.*

bioRxiv preprint doi: https://doi.org/10.1101/2023.12.13.571314; this version posted December 14, 2023. The copyright holder for this preprint (which was not certified by peer review) is the author/funder. This article is a US Government work. It is not subject to copyright under 17 USC 105 and is also made available for use under a CC0 license.

**Session Comparison**	**Hemisphere/Hand**	**Finger**	**Correlation Coefficient** (r) (mean ± std)
Pre to 3m	R/L	D1	0.83 ± 0.08
Pre to 3m	R/L	D2	0.84 ± 0.10
Pre to 3m	R/L	D3	0.88 ± 0.06
Pre to 3m	R/L	D4	0.90 ± 0.04
Pre to 3m	R/L	D5	0.89 ± 0.04
Pre to 6m	R/L	D1	0.85 ± 0.07
Pre to 6m	R/L	D2	0.84 ± 0.07
Pre to 6m	R/L	D3	0.89 ± 0.05
Pre to 6m	R/L	D4	0.90 ± 0.04
Pre to 6m	R/L	D5	0.89 ± 0.04
Pre to 3m	L/R	D1	0.80 ± 0.12
Pre to 3m	L/R	D2	0.79 ± 0.13
Pre to 3m	L/R	D3	0.83 ± 0.12
Pre to 3m	L/R	D4	0.87 ± 0.07
Pre to 3m	L/R	D5	0.88 ± 0.07
Pre to 6m	L/R	D1	0.78 ± 0.16
Pre to 6m	L/R	D2	0.79 ± 0.13
Pre to 6m	L/R	D3	0.84 ± 0.10
Pre to 6m	L/R	D4	0.87 ± 0.08
Pre to 6m	L/R	D5	0.87 ± 0.07

Supplementary Table 1. Pearson correlations for controls finger representations across sessions.

Supplementary Video 1. Patient's motor control before amputation and phantom hand movements after amputation.
